# Investigating the role of exosomal long non-coding RNAs in drug resistance within female reproductive system cancers

**DOI:** 10.3389/fcell.2025.1485422

**Published:** 2025-01-24

**Authors:** Nooshafarin Shirani, Neda Abdi, Matin Chehelgerdi, Hajar Yaghoobi, Mohammad Chehelgerdi

**Affiliations:** ^1^ Clinical Biochemistry Research Center, Basic Health Sciences Institute, Shahrekord University of Medical Sciences, Shahrekord, Iran; ^2^ Novin Genome (NG) Lab, Research and Development Center for Biotechnology, Shahrekord, Iran; ^3^ Young Researchers and Elite Club, Shahrekord Branch, Islamic Azad University, Shahrekord, Iran

**Keywords:** exosomes, long non-coding RNAs, drug resistance, female reproductive system cancers, tumor microenvironment, chemotherapy efficacy, targeted therapies

## Abstract

Exosomes, as key mediators of intercellular communication, have been increasingly recognized for their role in the oncogenic processes, particularly in facilitating drug resistance. This article delves into the emerging evidence linking exosomal lncRNAs to the modulation of drug resistance mechanisms in cancers such as ovarian, cervical, and endometrial cancer. It synthesizes current research findings on how these lncRNAs influence cancer cell survival, tumor microenvironment, and chemotherapy efficacy. Additionally, the review highlights potential therapeutic strategies targeting exosomal lncRNAs, proposing a new frontier in overcoming drug resistance. By mapping the interface of exosomal lncRNAs and drug resistance, this article aims to provide a comprehensive understanding that could pave the way for innovative treatments and improved patient outcomes in female reproductive system cancers.

## 1 Introduction

Neoplasms affecting the female reproductive organs, such as ovarian, cervical, and endometrial types, constitute a substantial global health challenge due to their high incidence and associated mortality (Bertone et al., 2004). These malignancies are distinguished by distinct biological characteristics, causes, and treatment responses (Carninci et al., 2005). Despite the progress in medical research, the outlook for individuals with late-stage reproductive neoplasms remains unfavorable, primarily due to emerging drug resistance. Deciphering the underlying mechanisms of this resistance is crucial for improving treatment efficacy and extending patient survival (Li et al., 2019a).

Exosomes, small extracellular vesicles derived from endosomes, have emerged as key players in intercellular communication (Zhang et al., 2018). They carry a diverse range of molecular constituents, including proteins, lipids, DNA, mRNA, and various non-coding RNAs. In the field of oncology, exosomes contribute to numerous processes such as tumor progression, metastasis, immune response modulation, and the development of drug resistance (Lakhotia et al., 2020). The role of exosomes in the tumor milieu and their potential as biomarkers and therapeutic agents have become focal points in cancer research (O’Brien et al., 2020).

Long non-coding RNAs (lncRNAs), a class of non-coding RNAs that exceed 200 nucleotides in length and are not predominantly involved in protein synthesis, were initially perceived as transcriptional noise (Bertone et al., 2004; Carninci et al., 2005). However, they are now recognized for their critical roles in gene expression regulation at several levels, including chromatin reconfiguration, transcription, and post-transcriptional processing. In cancer research, lncRNAs are known to play essential roles in regulating oncogenic and tumor suppressive pathways (Li et al., 2019a; Zhang et al., 2018). The discovery of lncRNAs in exosomes has opened new avenues for understanding drug resistance in cancer. These exosomal lncRNAs, which can be transferred among cancer cells and other cells within the tumor microenvironment, influence drug sensitivity and resistance (Bertone et al., 2004; Carninci et al., 2005). The mechanisms by which exosomal lncRNAs contribute to drug resistance are multifaceted, involving the modulation of cell death, drug efflux, DNA repair processes, and interactions in the tumor microenvironment (Lakhotia et al., 2020).

A primary obstacle in treating cancers of the female reproductive system is the onset of resistance to established chemotherapy protocols. This resistance, which can be inherent or acquired, frequently results in treatment failure and cancer progression. Investigating exosomal lncRNAs presents a promising approach to comprehend and potentially counteract this resistance. These entities may act as indicators for predicting therapeutic response and as novel targets to increase the susceptibility of cancer cells to treatments. Recent investigations are beginning to decode the intricate connections between exosomal lncRNAs and mechanisms of drug resistance in cancers of the female reproductive system. Though still in early stages, this research offers significant potential for devising new therapeutic approaches. The future trajectory in this domain involves a thorough analysis of exosomal lncRNAs, understanding their biological roles, and assessing their clinical application.

## 2 The basics of exosomes

**FIGURE 1:**
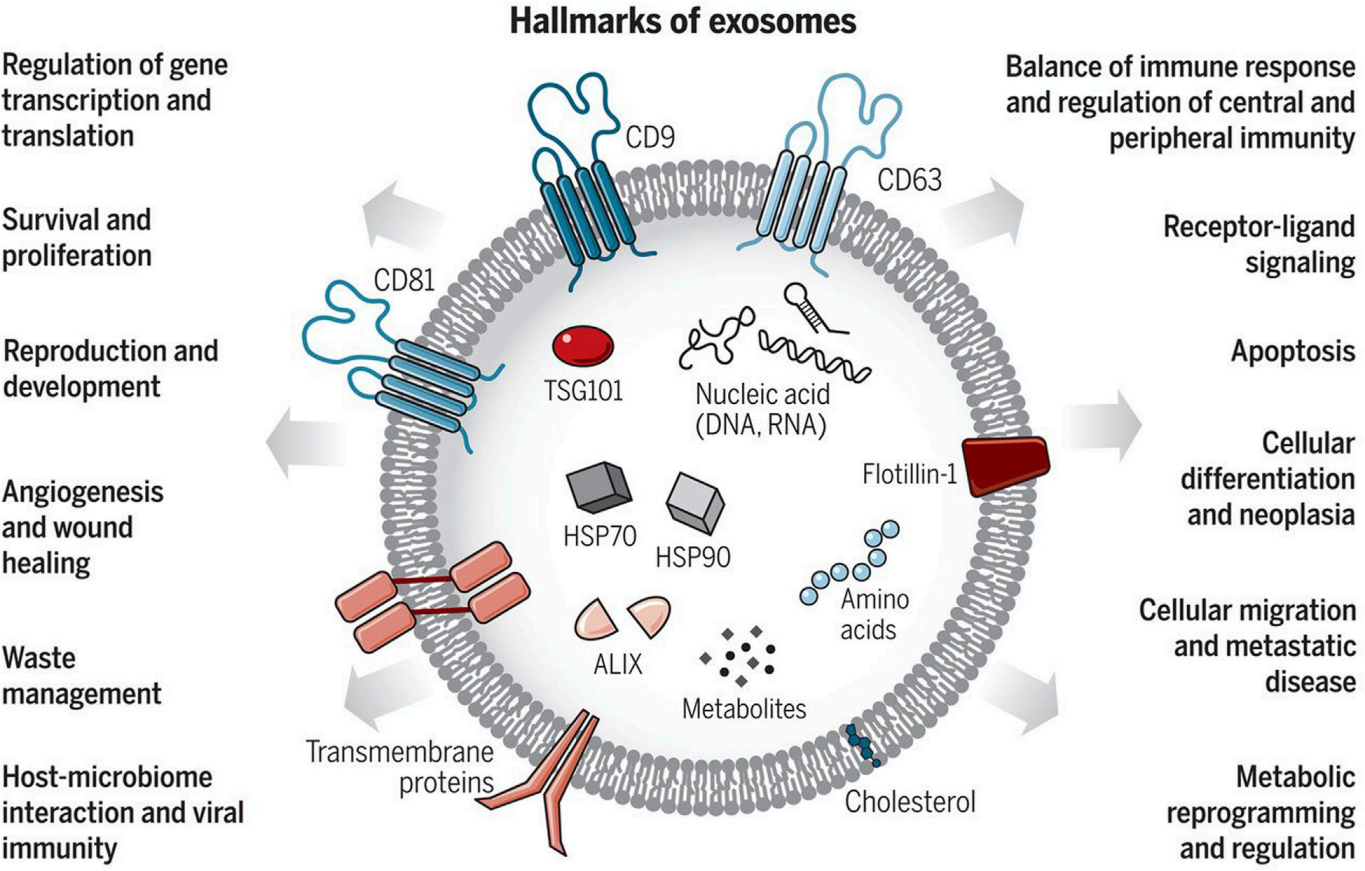
Exosomes: Sorting of Cellular Transport Network in Humans with Multifaceted Roles. Exosomes are tiny vesicles released by every cell type, encapsulating nucleic acids, proteins, lipids, and metabolic products. Serving as conduits for both paracrine and autocrine cell communication, they play critical roles in both healthy and diseased states, influencing numerous cellular processes. Re-printed from Science (Chang et al., 2021).

Exosomes are a subpopulation of extracellular vesicles (EVs), nanoscale structures with a lipid bilayer membrane performing their biological functions mainly by transferring cargoes such as proteins, RNAs, DNAs, and lipids in an intercellular communication substrate (Figure 1). Based on their size, EVs are divided into four groups: exosomes, microvesicles, apoptotic bodies, and oncosomes (O’Brien et al., 2020). In cancer biology, among EVs, exosomes play the most important regulatory role (Chang et al., 2021; De Los Santos et al., 2019; Fontana et al., 2021). The term exosome (different from exosome complex, which plays a role in RNA degradation) was first applied to vesicles of unknown origin released from cultured cells with 5′-nucleotidase activity (van Niel et al., 2018), and they were thought to contain cellular waste (Harding and Stahl, 1983; Pan and Johnstone, 1983). Exosomes usually differ in size between 40 and 150 nm, and their surface contains marker proteins such as tetraspanins (CD63, CD81, CD82, and CD9), flotillin, and MHC, depending on their origin cell (Zhang et al., 2018; Hao et al., 2022). Generally, exosome biogenesis occurs within the endosomal system. Several steps of this process are regulated by intracellular and extracellular signals. At the start, the invagination of the plasma membrane forms a primary endosome, and the maturation of it goes on with continuous buddying of intraluminal vesicles (ILVs) into the primary endosome space. The primary endosome containing ILVs is known as the primary multivesicular body (MVB) at this stage (De Los Santos et al., 2019; Bebelman et al., 2018; Liu et al., 2022a). Two pathways have been identified in the process of ILV generation. One way requires ESCRT, a cluster of five subunits (ESCRT-0, ESCRT-I, ESCRT-II, ESCRT-III, and Vps4), and the other one is referred to as ESCRT-independent. It has been observed that the ESCRT-dependent pathway can actually be considered as the main pathway. There are also some other pathways dependent on ceramide or tetraspanins, which fall under the ESCRT-independent category. For example, nSMase2 produces ceramide via hydrolysis of sphingomyelin located in the MVB membrane, thus, playing a role in endosomal maturation (Fu et al., 2019; Trajkovic et al., 2008). In this stage, exosome cargoes are loaded into the exosome, and then transmembrane proteins such as tetraspanins are added to the exosome membrane, which means endosomal sorting is completed (van Niel et al., 2011). After that, the MVB may be destroyed via the lysosomal pathway or is converted into a mature MVB for exosomal release, which happens with the help of cytoskeleton proteins such as actin and microtubules (De Los Santos et al., 2019; Mashouri et al., 2019). Finally, Rab GTPase proteins such as Rab27a and Rab27b, which control vesicular transport, enable MVB binding and exosome release into the extracellular space (Mathieu et al., 2019; Ostrowski et al., 2010). Also, Rab27a and Rab27b have been shown to regulate the trafficking of the pro-invasive matrix metalloproteinase MMP14 (Macpherson et al., 2014). This process is aided by the SNARE complex that is responsible for MVB and cell membrane fusion (Hessvik and Llorente, 2018).

### 2.1 Role of exosomes in cell-cell communication

**FIGURE 2:**
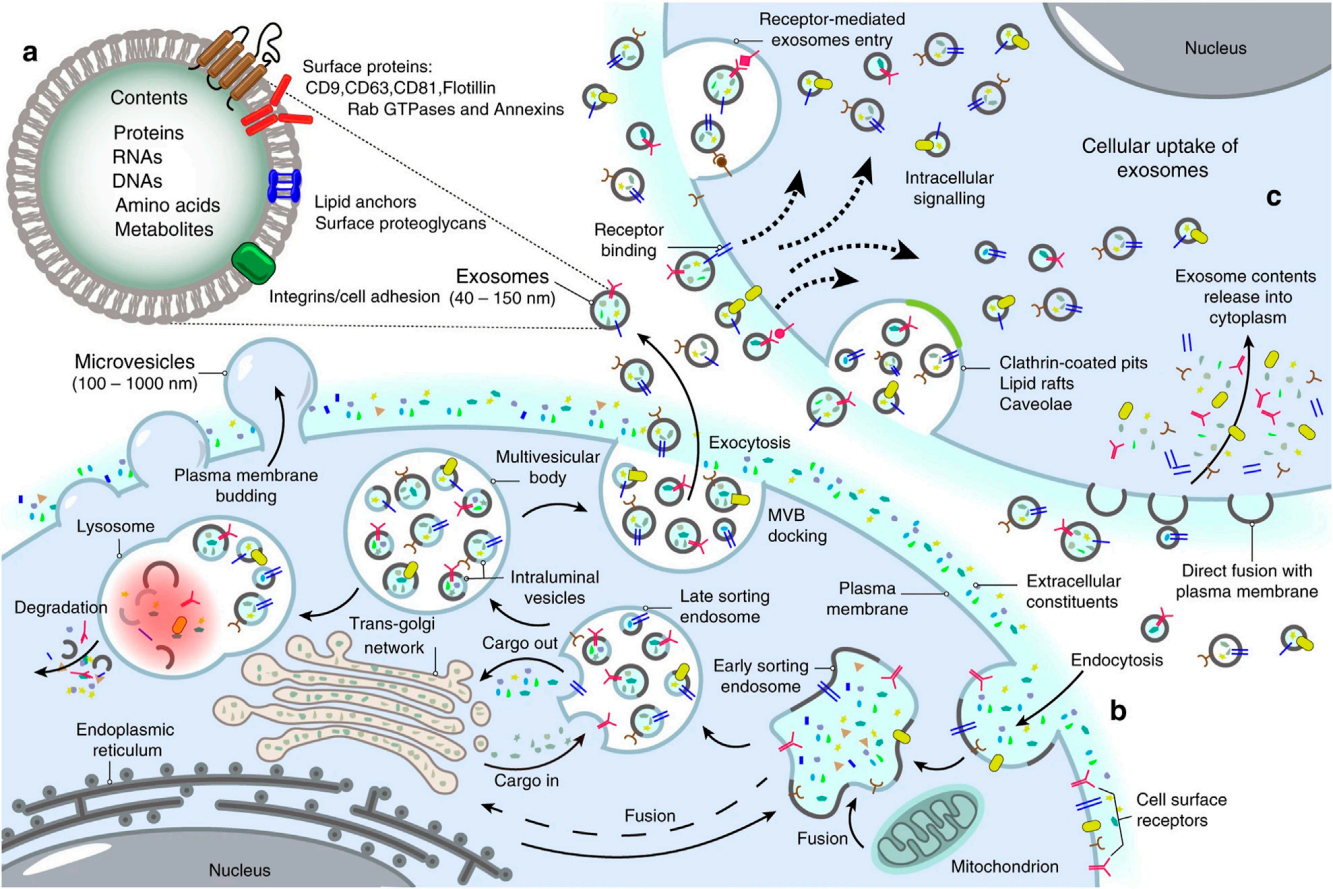
Landscape of exosome biogenesis. **(A)** Exosomes are composed of various proteins, nucleic acids, amino acids, and metabolites. Specific markers such as CD9, CD63, CD81, flotillin, and Annexins help identify them. **(B)** The process begins with the uptake of extracellular elements and cell surface proteins through endocytosis and invagination of the plasma membrane. This leads to the creation of early sorting endosomes (ESEs) through the merging of plasma membrane buds with components from the endoplasmic reticulum (ER), trans-Golgi network (TGN), and mitochondria. ESEs evolve into late sorting endosomes (LSEs), where further invagination and cargo modification result in the production of intraluminal vesicles (ILVs) and the development of multivesicular bodies (MVBs). Some MVBs merge with lysosomes for degradation of their contents, while others are transported to the cell surface, where they fuse with the plasma membrane. This fusion leads to the release of ILVs as exosomes outside the cell. **(C)** Exosomes can then enter other cells through various mechanisms, including direct fusion with the plasma membranes, receptor-mediated uptake, clathrin-coated pits, and lipid rafts. Re-printed from Springer Nature (De Los Santos et al., 2019).

Exosomes possess the ability to transport molecular cargos from a donor cell to a recipient cell; thus, they are considered a major piece of cell-cell communications (Hannafon and Ding, 2013). Dendritic cells, macrophages, cancer cells, and mesenchymal stem cells are only some of the cell types utilizing this mechanism as a means of local and systematic communication. Figure 2 describes the process of exosome biogenesis. As the cargos they carry to new cells are capable of exerting new biological changes, these exosomes can affect various mechanisms of cell biology in health and disease (Kalluri and LeBleu, 2020; Gurung et al., 2021). Table 1 outlines the crucial functions of exosomes in facilitating cell-cell communication, a fundamental process in both physiological and pathological contexts. After exosomes are released into the extracellular matrix (ECM), they proceed to their destination cell by taking one of the following pathways: autocrine, juxtacrine, paracrine, or endocrine (Hao et al., 2022). Uptake of exosomes is done depending on the cell they enter. Exosomes may bind directly to the surface receptors of a recipient cell through their surface ligands, such as glycans, lectins, integrins, or other cell adhesion molecules. This results in the activation of the downstream signaling pathway without internalization. In the second approach, exosome uptake is done through endocytosis, including clathrin-dependent endocytosis, clathrin-independent endocytosis, phagocytosis, or macropinocytosis. After exosomes enter recipient cells, endosomes containing them may be degraded within lysosomes, recycled through plasma membrane recombination, or released into target cells (Hessvik and Llorente, 2018; Gurung et al., 2021; Han et al., 2020; McKelvey et al., 2015).

**TABLE 1:** Role of exosomes in cell-cell communication.

Exosome source	Molecular composition	Recipient cell type	Biological function	Mechanism of action	Clinical/Research implications	References
Dendritic Cells (Immune System)	MHC proteins, co-stimulatory molecules, cytokines, miRNAs	T lymphocytes, B lymphocytes	Antigen presentation, immune activation, immune suppression	Fusion with plasma membrane, receptor-mediated endocytosis	Development of vaccines, immunotherapies for cancer and autoimmune diseases	Montecalvo et al. (2008) and Li (2022)
Neural stem cells (Brain)	Growth factors, neurotrophic factors, miRNAs	Neurons, glial cells	Promote neuronal growth, regeneration, and survival	Fusion with plasma membrane, endocytosis	Treatment of neurodegenerative diseases, brain repair strategies	Yuan et al. (2021) and Zhong et al. (2023)
Tumor cells (Cancer)	Oncogenes, growth factors, miRNAs, immunosuppressive molecules	Endothelial cells, fibroblasts, other immune cells	Promote tumor growth, invasion, metastasis, immune evasion	Fusion with plasma membrane, endocytosis	Potential biomarkers for cancer diagnosis and prognosis, development of anti-cancer therapies	Kalluri and LeBleu (2020) and Whiteside (2016)
Mesenchymal stem cells (Bone marrow)	Growth factors, immunomodulatory molecules, miRNAs	Damaged tissues, immune cells	Tissue repair, regeneration, immunomodulation	Fusion with plasma membrane, endocytosis	Cell-based therapies for wound healing, inflammatory diseases	Phinney and Pittenger (2017) and Lai et al. (2013)
Epithelial cells (Intestine)	Digestive enzymes, immune molecules, microRNAs	Enterocytes, immune cells	Nutrient absorption, barrier function, immune regulation	Fusion with plasma membrane, endocytosis	Understanding gut health, potential treatments for inflammatory bowel diseases	van Niel et al. (2018) and Zhang et al. (2016)
Platelets (Blood)	Growth factors, clotting factors, inflammatory mediators	Endothelial cells, immune cells	Wound healing, hemostasis, inflammation	Fusion with plasma membrane, endocytosis	Development of novel wound healing therapies, understanding thrombotic disorders	Liu et al. (2019) and Nurden (2011)
Erythrocytes (Blood)	MicroRNAs, adhesion molecules	Endothelial cells, immune cells	Gas exchange, oxygen delivery, modulation of vascular function	Endocytosis, interaction with cell surface receptors	Potential biomarkers for red blood cell disorders, understanding sickle cell disease	Chen et al. (2008) and Kato et al. (2018)
Osteoclasts (Bone)	Collagenase, growth factors	Osteoblasts	Bone resorption, remodeling	Fusion with osteoblast membrane, receptor-mediated endocytosis	Treatments for osteoporosis, understanding bone diseases	Yang et al. (2020) and Li et al. (2016)
Cardiac fibroblasts (Heart)	Collagen, inflammatory mediators, miRNAs	Cardiomyocytes, immune cells	Cardiac fibrosis, inflammation, post-injury repair	Fusion with cell membrane, endocytosis	Potential treatments for heart failure, understanding cardiac remodeling	Hohn et al. (2021) and Reiss et al. (2023)
Endothelial cells (Blood vessels)	Angiogenic factors, adhesion molecules, miRNAs	Smooth muscle cells, immune cells	Vascular growth, inflammation, immune regulation	Fusion with plasma membrane, endocytosis	Understanding cardiovascular diseases, development of anti-angiogenic therapies	Huber and Holvoet (2015), and Yang et al. (2023)
Adipocytes (Fat tissue)	Lipases, inflammatory mediators, miRNAs	Skeletal muscle cells, liver cells	Lipid metabolism, insulin sensitivity, inflammation	Endocytosis, interaction with cell surface receptors	Understanding obesity and related metabolic disorders, development of anti-inflammatory therapies	Lei et al. (2021) and Mei et al. (2022)
Macrophages (Immune system)	Cytokines, chemokines, MHC proteins	T lymphocytes, B lymphocytes, other immune cells	Immune activation, antigen presentation, inflammation resolution	Fusion with plasma membrane, endocytosis, receptor-mediated interactions	Development of immunomodulatory therapies, understanding autoimmune diseases and infections	Gangadaran et al. (2023) and Hazrati et al. (2022)
Fibroblasts (Connective tissue)	Extracellular matrix proteins, growth factors, cytokines	Epithelial cells, endothelial cells, immune cells	Tissue repair, wound healing, inflammation	Fusion with plasma membrane, endocytosis, paracrine signaling	Development of regenerative medicine therapies, understanding wound healing processes	Manzoor et al. (2024) and Prasai et al. (2022)
Chondrocytes (Cartilage)	Collagen, proteoglycans, growth factors	Synovial fibroblasts, chondrocytes	Cartilage maintenance, repair, inflammation	Fusion with plasma membrane, endocytosis, interaction with extracellular matrix	Understanding osteoarthritis, development of cartilage repair strategies	Fan et al. (2022) and Kato et al. (2014)
Epithelial cells (Lung)	Surfactant proteins, antimicrobial peptides, miRNAs	Alveolar macrophages, immune cells	Gas exchange, barrier function, immune defense	Fusion with plasma membrane, endocytosis, paracrine signaling	Understanding lung diseases like asthma and COPD, development of novel therapeutic approaches	Liu et al. (2021) and Andres et al. (2020)
Muscle stem cells (Skeletal muscle)	Myogenic factors, growth factors, miRNAs	Muscle satellite cells, fibroblasts	Muscle regeneration, repair, growth	Fusion with plasma membrane, endocytosis, activation of signaling pathways	Development of cell-based therapies for muscle diseases, understanding muscle repair mechanisms	Porcu et al. (2024) and Luo et al. (2024)
Endocrine cells (Pancreas, thyroid)	Hormones, growth factors, miRNAs	Target tissues (e.g., liver, muscle, brain)	Metabolic regulation, cell growth, differentiation	Fusion with plasma membrane, endocytosis, receptor-mediated signaling	Understanding endocrine disorders like diabetes and thyroid diseases, development of targeted therapies	Wang et al. (2020) and Zou et al. (2021)
Tumor-associated macrophages (Cancer)	Growth factors, immunosuppressive molecules, miRNAs	Tumor cells, immune cells	Promote tumor growth, metastasis, immune evasion	Fusion with plasma membrane, endocytosis, paracrine signaling	Understanding tumor microenvironment, development of immunotherapies targeting TAMs	Zhou et al. (2023) and Panda et al. (2024)
Neutrophils (Immune system)	Neutrophil extracellular traps (NETs), antimicrobial peptides, cytokines	Pathogens, other immune cells	Defense against pathogens, inflammation resolution	Direct interaction with pathogens, endocytosis by immune cells	Understanding neutrophil-mediated immunity, development of new antimicrobial strategies	Gong et al. (2024), and Gangadaran et al. (2023)

### 2.2 Molecular contents of exosomes: roteins, lipids, and RNAs

**FIGURE 3:**
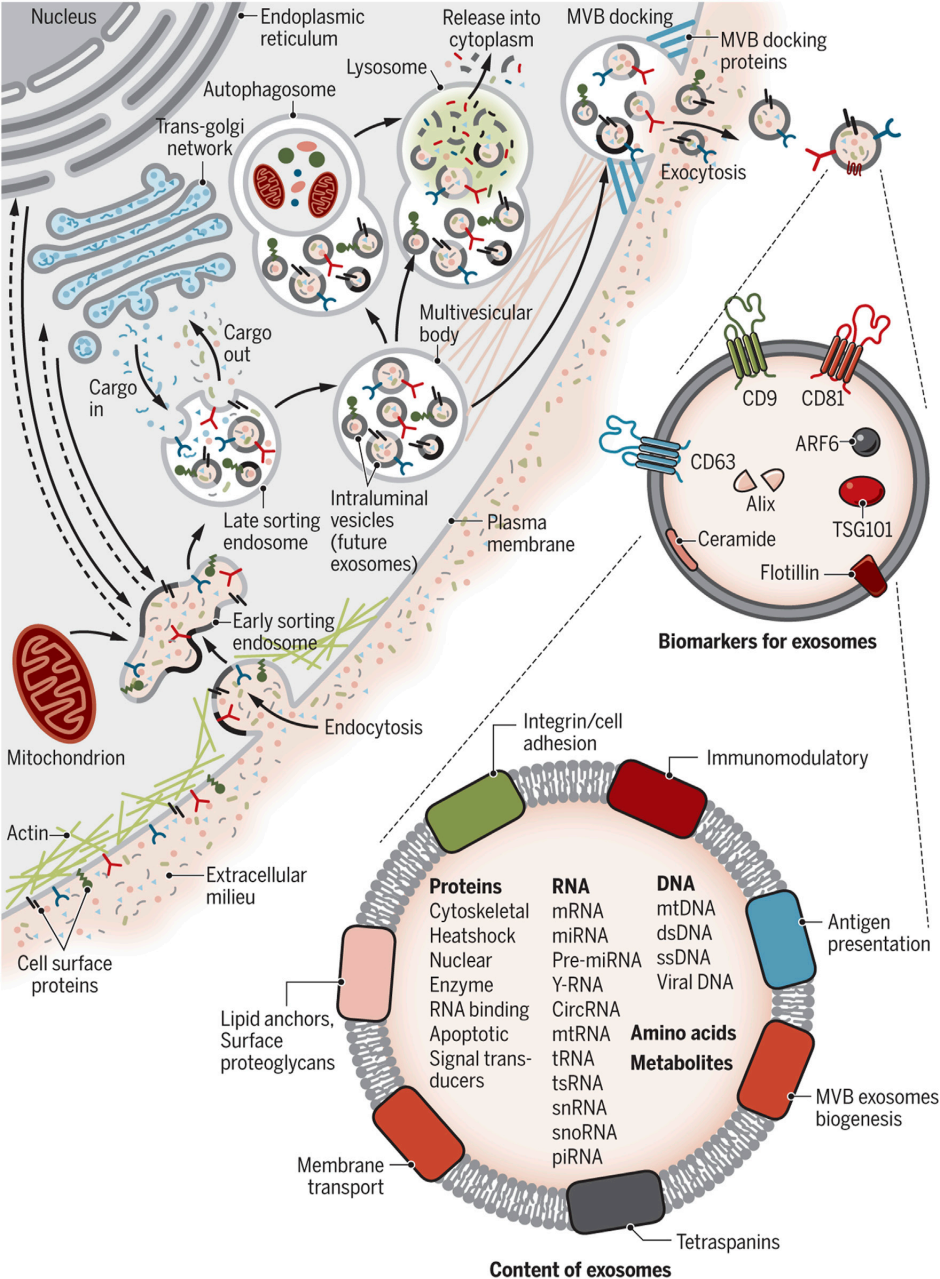
Exosome and cargo recycling. The process begins when various substances, including proteins, lipids, metabolites, and small molecules, enter cells through endocytosis or the inward folding of the cell membrane. This leads to the creation of early sorting endosomes (ESEs), either directly through budding or by merging with pre-existing ESEs formed from components of the endoplasmic reticulum (ER), trans-Golgi network (TGN), and mitochondria. These ESEs can merge with the ER and TGN, suggesting a method for the endocytic materials to reach these organelles. ESEs, containing a mix of membrane and internal materials from various origins, evolve into late sorting endosomes (LSEs). A second folding within the LSE forms intraluminal vesicles (ILVs), altering the composition of what will become exosomes, including the entry of cytoplasmic elements into these vesicles. This process can distribute cell surface proteins across ILVs in a unique manner and lead to ILVs of different sizes and compositions. LSEs then develop into multivesicular bodies (MVBs) that contain these ILVs. MVBs may merge with autophagosomes for content degradation in lysosomes, or directly with lysosomes. Alternatively, MVBs can move to the cell surface, where they release exosomes through exocytosis, maintaining a similar membrane orientation. The creation of exosomes involves several proteins, such as Rab GTPases and ESCRT proteins, along with markers like CD9, CD81, CD63, and others. Exosomes are characterized by their content, which includes various proteins, RNAs, DNAs, amino acids, and metabolites, as well as surface proteins like tetraspanins and integrins. Re-printed from Science (Chang et al., 2021).

Figure 3 describes the exosome and cargo recycling. Exosomes may partake in transferring various types of functional molecules such as lipids, proteins, and genetic material, and have been identified in different body fluids, including saliva, urine, breast milk, semen, blood, and bronchoalveolar lavage fluid (van Niel et al., 2011; Wang et al., 2016). These vesicles may play a different role based on their donor cell, their destination, or the cargos they carry. In addition, they can transfer bioactive molecules between cancer and other cells in the tumor microenvironment over near or long distances and are involved in cancer development by controlling processes such as immune system regulation, metastasis, angiogenesis, epithelial-mesenchymal transition (EMT), entry into quiescence (G0 phase), senescence, and drug resistance (Li, 2022; Soltész et al., 2021; Sundararajan et al., 2018; Tai et al., 2018). The fact that drug resistance can be induced in drug-sensitive cells through exosomes derived from exosome-resistant cells has been approved in several studies (Li and Nabet, 2019; Lobb et al., 2017; Mikamori et al., 2017; Takahashi et al., 2014a). Increasing the expression of multidrug resistance proteins, expelling chemotherapeutic drugs from cells, decreasing drug uptake, drug detoxification, and increasing DNA repair are some of the mechanisms through which exosomes increase drug resistance, all by releasing bioactive molecules in their target cells (Li, 2022; Sundararajan et al., 2018; Guo et al., 2020a; Li et al., 2020; Xie et al., 2019). Interestingly, some mediators of drug resistance, such as annexin 3 and RAB7, have been proven to participate in production of EVs in cancer cells. This means drug-resistant cells are eager to cause resistance in non-resistant cells (Guerra and Bucci, 2019; Xavier et al., 2020; Yin et al., 2012).

## 3 Understanding long non-coding RNAs

Complete sequencing of the human genome and developing technologies such as high-throughput next-generation sequencing have aided our understanding of coding and non-coding RNAs (Bertone et al., 2004; Carninci et al., 2005). About 75% of the human genome is transcribed into RNA, and of this amount of RNA, only 3% contains protein-coding mRNA (Yan and Bu, 2021). Non-coding RNAs (ncRNAs) can be classified into different types based on their transcript length, function, structure and position in the genome (Lakhotia et al., 2020). The ncRNAs that play an important role in regulating gene expression and have been studied in recent years include small interfering RNAs (siRNAs), microRNAs (miRNAs), Piwi-interacting RNAs (piRNAs), circular RNAs (circRNA), and long ncRNAs (lncRNA) (Yan and Bu, 2021). According to a classification based on transcript size, ncRNAs are divided into two groups: small ncRNAs (less than 200 nt) and long ncRNAs (more than 200 nt) (Kashi et al., 2016; St Laurent et al., 2015). ciRNAs and lncRNAs are both composed of more than 200 nucleotides, and the difference lies in their structure. LncRNAs are generally linear molecules that can form complex seconary structures, such as stem-loops and hairpins, while circRNAs are characterized by a covalently closed loop, lacking 5′ and 3′ ends (Yan and Bu, 2021; Zampetaki et al., 2018). LncRNAs are mRNA-like transcripts longer than 200 nt with limited protein-coding potential (Gao et al., 2020; Sahu et al., 2015). Most of them are transcribed by the RNA polymerase II and have a polyadenylated 3′ end and a m7G-cap at their 5′ end (Statello et al., 2021). Unlike mRNA, lncRNAs usually lack an open reading frame, have fewer exons, and are also expressed at lower levels with more tissue-specific expression patterns. These types of ncRNAs have been under weaker pressure of natural selection during evolution, and many of them are specific to mammals (Li et al., 2019a; Zhang et al., 2018). LncRNAs are classified into different classes according to their location in the genome, sequence, length, morphology, structure, and functional properties. Based on their genomic position, there are six general groups: intergenic (between two protein-coding genes), intronic (in the intronic regions of the protein-coding gene), enhancer (eRNA; transcribed from the DNA sequence of the enhancer regions), sense (transcribed from the same strand and in the same direction as the neighboring protein-coding gene), antisense (transcribed from the opposite strand and in the opposite direction of the neighboring protein-coding gene), and bidirectional (1 Kb away from the promoter region of a protein-coding gene, and it is transcribed from the opposite strand) (Hermans-Beijnsberger et al., 2018; Blokhin et al., 2018; Sun and Kraus, 2015; Borkiewicz et al., 2021). Table 2 outlines the diverse roles and mechanisms through which long non-coding RNAs (lncRNAs) are involved in biological functions.

**TABLE 2:** Biological Functions and Mechanisms of lncRNAs.

lncRNA name/Identifier	Model systems	Expression profile	Functional role	Interaction partners	References
LincRNA-p21 (ENSMUSG00000085912)	In vivo (mouse models)/In vitro (Rosa26-CreER^T2^ MEFs and p21^+/−^ MEFs cells)	Low expression in proliferating cells	Regulates cell cycle	p21 protein, hnRNP-K	Huarte et al. (2010) and Dimitrova et al. (2014)
KCNQ1OT1 (ENSG00000269821)	In vivo (mouse models)/In vitro (MEFs and JEG-3 cells)	Imprinted gene, expressed in maternal allele	Fetal development	G9a, PRC2 complex, Dnmt1	Pandey et al. (2008) and Mohammad et al. (2010)
ZFAS1 (ENSG00000177410)	In vitro (HC11, T47D, BT474, MCF7, and N2A cells)	High expression in the mammary gland, low expression in breast tumors	alveolar development and epithelial cell differentiation in the mammary gland	Snord12, Snord12b, and Snord12c	Askarian-Amiri et al. (2011)
SNHG16 (ENSG00000163597)	In vitro (DLBCL, OCI‐LY7)	High expression in multiple cancers	Promotes tumorigenesis and metastasis	miR‐497‐5p, MYC, EZH2	Zhu et al. (2019) and Xiao et al. (2020a)
GATA3-AS1 (ENSG00000197308)	*In vitro* (human PBMC)	High expression in TH2 polarizing conditions, high expression in MS patients	Efficient transcription of GATA3, IL-4, IL-5, and IL-13	GATA3	Gibbons et al. (2018) and Keshavarz et al. (2024)
	In vivo (MS patients and healthy individuals)				
NEAT1 (ENSG00000245532)	In vivo (mouse models)/In vitro (HeLa, SH-SY5Y, and U2OS cells)	High expression in nuclear paraspeckles	Scaffolding for paraspeckle assembly, enlarges paraspeckles, gene expression regulation	PSF, SFPQ, paraspeckle proteins	Hirose et al. (2014), An et al. (2022), and Naganuma et al. (2012)
AIRN (ENSG00000268257)	In vivo (mouse models)	Imprinted gene, expressed in maternal allele	Blocks chromosome interactions, initiates paternal-specific silencing	Slc22a3 promoter chromatin, G9a	Nagano et al. (2008), Andergassen et al. (2019), and Santoro et al. (2013)
BARD1 (ENSG00000138376)	In vitro (HEK293T, HeLa, NuTu-19, and human prostate cancer cells)	Ubiquitous, high expression in brain and heart	Regulates cell differentiation and apoptosis	p53, BRCA1	Feki et al. (2005) and Irminger-Finger and Jefford (2006)
Braveheart (ENSMUSG00000098098)	*In vitro* (ES cells)	Cardiac-specific expression	Cardiac development	MesP1, Gata4, Hand1, Hand2, Nkx2.5, Tbx5	Klattenhoff et al. (2013) and Devaux et al. (2015)
SLIRP (ENSG00000119705)	*In vitro* (MCF-7, MDA-MB-468, SK-BR-3)/*In vivo* (*Drosophila*)	Ubiquitous	Regulates mitochondrial function	SRA, SLIRP, SRC-1, and NCoR	Hatchell et al. (2006)
lncRNA-JADE (ENSG00000280711)	*In vivo* (mouse models)/*In vitro* (NIH3T3 and MCF7 cells)	High expression in breast cancer tissues	Connects the DNA damage response (DDR) to histone H4 acetylation	Brca1	Wan et al. (2013)
GAS6-AS1 (ENSG00000233695)	*In silico*/*In vitro* (tissue samples, human AML cells)	Low expression in AF related stroke (AFST), High expression in AML	Downregulates GOLGA8A and BACH2	YBX1, MYC	Li et al. (2023) and Zhou et al. (2021a)
			Upregulates MYC target genes associated with leukemia progression		
Evf2 (ENST00000492509)	*In vivo* (mouse models)	Specific expression in certain brain regions and developmental stages	Regulates gene distances and cohesin binding in the mouse embryonic forebrain	Sox2, Dlx5/6UCE	Cajigas et al. (2021) and Cajigas et al. (2018)
HULC (ENST00000642163.1)	*In vivo* (liver and bladder cancer patients)/*In vitro* (HL-7702)/	High expression in liver, gastric, and bladder cancers	Promotes tumorigenesis and inhibits apoptosis	miR-372, miR-9-5p, MYH9, ZIC2	Wang et al. (2010a), Tan et al. (2020), and Wang et al. (2017)
MALAT1 (ENSG00000251562)	*In vitro* (HeLa cells)	Ubiquitously expressed	Regulates alternative splicing	SR splicing factors, nuclear proteins	Tripathi et al. (2010)

### 3.1 The biological functions and mechanisms of lncRNAs

**FIGURE 4:**
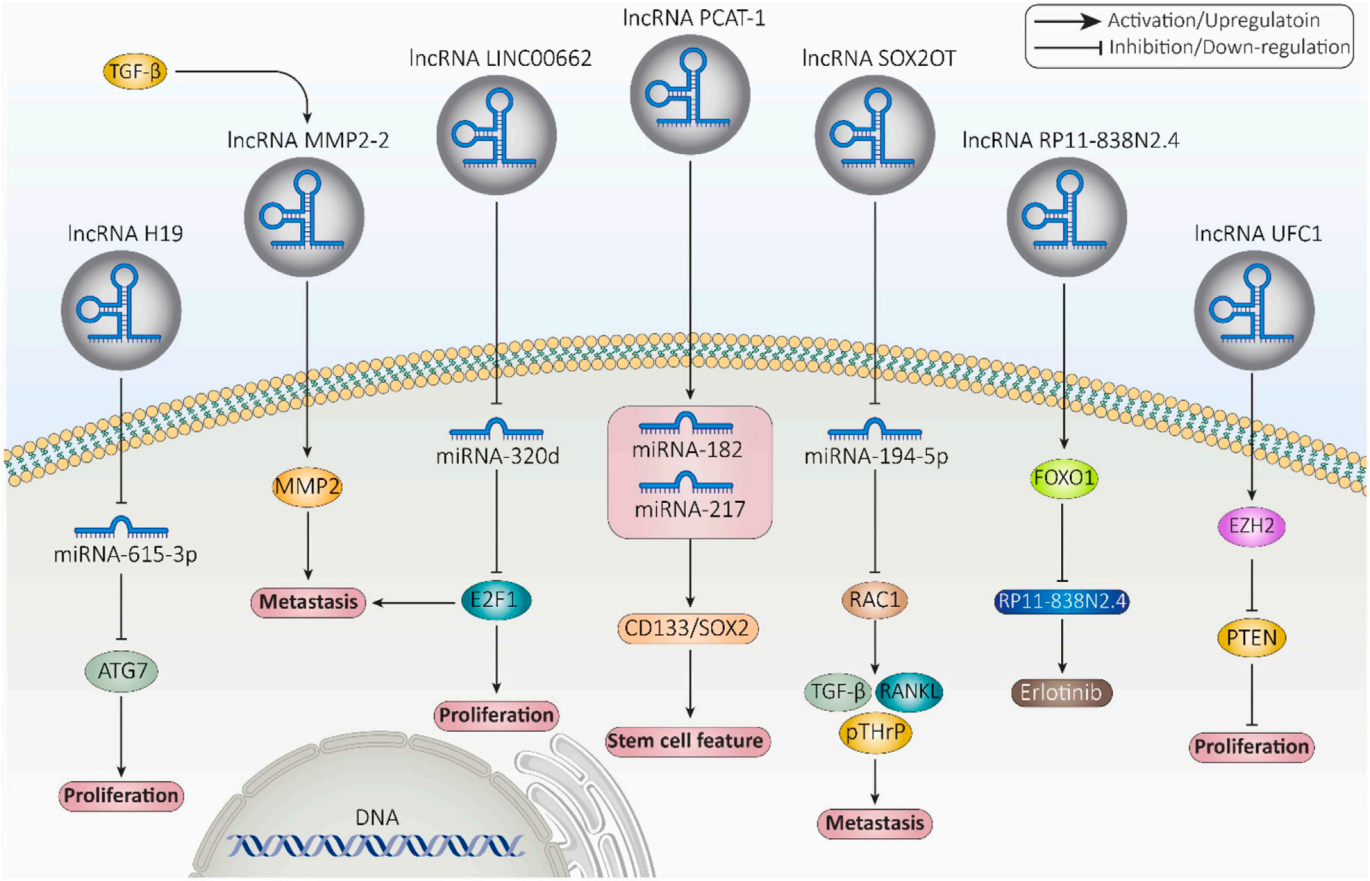
Examples of long non-coding RNAs (lncRNAs) and their mechanisms involved in cancer progression (Fontana et al., 2021).

LncRNAs can be observed in a variety of intracellular components such as the nucleus or cytoplasm (Gudenas and Wang, 2018). LncRNAs are capable of modifying a landscape of cellular process, such as replication, pre/post transcription, and translation. They are able to regulate chromatin structure at various functional stages prior to transcription, which include histone methylation and acetylation, DNA methylation, and chromatin remodeling (Li et al., 2019a; Liu et al., 2022b). For example, the lncRNA HOTTIP binds to the WDR5-MLL complex and then, by targeting the ΄5HOXA locus, mediates activation of HOXA transcription through H3K4 methylation (Wang et al., 2011). In addition, lncPRESS1 plays an important role in regulating the cell differentiation process through its interaction with the deacetylase SIRT6 (Jain et al., 2016). Furthermore, lncRNAs can play the role of a cis-regulator or a trans-regulator, or they can interfere with imprinting in pre-transcriptional regulation (Sun and Kraus, 2015; Jarroux et al., 2017; Ma et al., 2013; Wang et al., 2021a; Yao et al., 2019; Kornienko et al., 2013). LncRNAs can also regulate gene expression by binding directly to transcription factors or the polymerase machinery or by interfering with polymerase-promoter bonds (He et al., 2019). For example, the lncRNA 7SK represses elongation by binding to the transcription factor PTEFβ and preventing its phosphorylation for elongation (Peterlin et al., 2012). The mechanisms of lncRNAs interfering with post-transcriptional regulation include regulation of mRNA alternative splicing, mRNA stability, protein stability, DNA regulation, regulation of protein localization, and acting as a sponge for miRNA (Li et al., 2019a; Statello et al., 2021; Pisignano and Ladomery, 2021; Karakas and Ozpolat, 2021; Zhou et al., 2019). For instance, the lncRNA TINCR interacts with the staufen1 (STAU1) protein to promote the stability and expression of differentiation-related mRNAs such as KRT80 (Kretz et al., 2013). Figure 4 provides examples of long non-coding RNAs (lncRNAs) and their mechanisms that contribute to cancer progression.

### 3.2 lncRNAs in disease and cancer progression

Regarding the biological functions of lncRNAs and their significant contribution to both activation and inhibition of gene expression, lncRNAs have the potential to participate in various diseases such as cancer (Ahadi, 2021). According to multiple studies, dysregulation of lncRNAs may contribute to cancer initiation and development by affecting different biological processes and pathways such as proliferation, differentiation, metastasis, invasion, cell death, angiogenesis, cell cycle, and miRNA silencing (Ahadi, 2021; Smolarz et al., 2021). LncRNA SChLAP1 acts as an antagonist of SWI/SNF chromatin remodeling complex in prostate cancer cells and contributes to metastasis and aggressiveness of cancer cells (Prensner et al., 2013). TFAP2A-AS1, which is associated with decreased expression in breast cancer, acts as a miRNA sponge for miR-933 and this way regulates Smad2 expression (Zhou et al., 2019). Among the lncRNAs related to various cancers are MALAT1, HOTAIR, LUCAT1, MRPS30-DT, and IFNG-AS1 (Taniue and Akimitsu, 2021; Xing et al., 2021; Hajjari and Salavaty, 2015; Shirani et al., 2024; Yaghoobi et al., 2018). Therefore, lncRNAs can be used in diagnosis and targeted therapy in cancer.

## 4 Exosomal lncRNAs: the interface

There is still no detailed understanding of how lncRNAs are packaged into exosomes. However, studies conducted have been suggesting that several proteins, including the heterogeneous nuclear ribonucleoproteins (hnRNP) family, such as hnRNPA2B1 (Chen et al., 2020a; Lei et al., 2018; Zheng et al., 2019) and hnRNPK (Gao et al., 2018) as well as human antigen R (HuR) (Deng et al., 2020) are some of the proteins that aid the sorting of lncRNAs into exosomes (Fabbiano et al., 2020). What is clear is that in almost every situation, RNAs are present as a ribonucleoprotein (RNP) complex in cells or exosomes. Thus, proteins with the ability of shaping a complex with RNAs are undoubtedly essential for the encapsulation of ncRNAs into exosomes (Zang et al., 2020).

**FIGURE 5:**
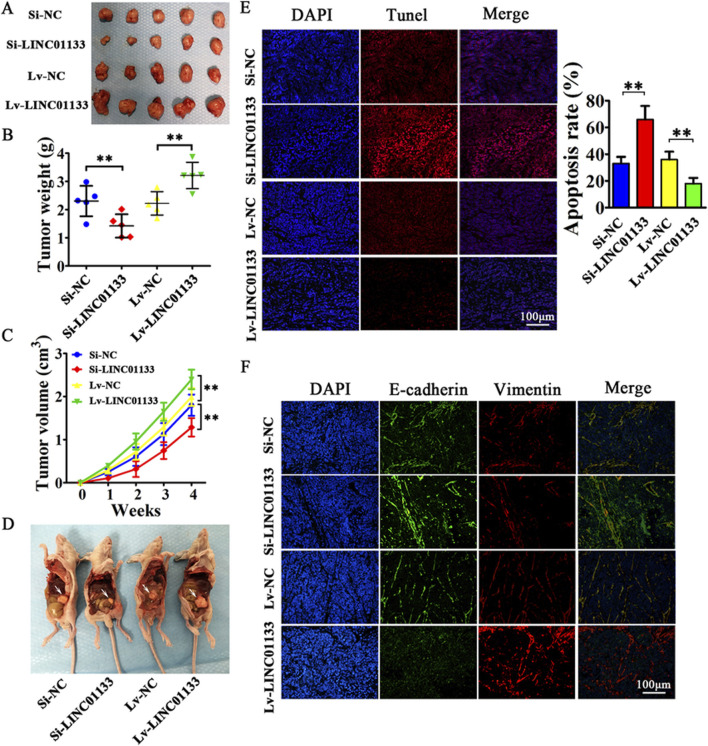
the results of an experiment where 4 million CFPAC-1 cells, treated with either Si-LINC01133, Si-NC, Lv-LINC01133, or Lv-NC, were injected into the right side of nude mice. **(A)** One month later, the mice were euthanized, and the tumor grafts were removed for analysis. **(B)** The findings showed that CFPAC-1 cells treated with Lv-LINC01133 resulted in heavier xenografts, while those treated with Si-LINC01133 led to lighter xenografts. **(C)** Additionally, CFPAC-1 cells exposed to Lv-LINC01133 demonstrated faster growth and larger tumor volumes compared to those treated with Si-LINC01133, which exhibited slower growth and smaller tumor volumes. **(D)** Furthermore, when 1 million CFPAC-1 cells treated with each of the substances were injected into the lower left abdomen quadrant, the spread within the abdominal cavity was assessed, with metastatic nodules identified by white arrowheads. **(E)** The study also found that LINC01133 significantly reduced apoptosis in the xenograft tumors of CFPAC-1 cells, as verified by TUNEL assay, with the apoptosis rate measured across five random fields in three repeated experiments. **(F)** Lastly, the expression levels of E-cadherin and Vimentin in the xenograft tumors were examined through immunofluorescence. Re-printed from Springer Nature (van Niel et al., 2018).

One protein whose role in regulating lncRNAs has been proven is hnRNPA2B1 (Qiu et al., 2021). In bladder cancer (BC) cells, hnRNPA2B1 specifically binds to lncRNA LNMAT2 and is packed into exosomes. This was approved because hnRNPA2B1 knockdown had no effect on the expression levels of LNMAT2 in BC cells, but exosome levels of this lncRNA was evidently decreased (Chen et al., 2020a). LncRNA H19 in human non-small cell lung cancer (NSCLC) also forms a complex with hnRNPA2B1. The investigation by Lei et al. further proved that evaluated expression levels of hnRNPA2B1 promoted exosomal levels of H19 secreted by gefitinib-resistance cells of NSCLC (Lei et al., 2018). In another study conducted by Chen et al., lncARSR could bind to hnRNPA2B1 and be packaged into exosomes. hnRNPA2B1-lncARSR complex was seen in cytoplasm and exosomes rather than nucleus, which hnRNPA2B1 was initially identified. This observation proved that hnRNPA2B1 is specifically involved in the wrapping of lncARSR into the exosomes (Qu et al., 2016). Zheng et al. also indicated that in human breast cancer cells, hnRNPA2B1 is upregulated and has a correlation with the expression of exosomal lncRNA AGAP2AS1, and silencing hnRNPA2B1 resulted in downregulation of AGAP2AS1. Ultimately, it was proved that encapsulation of AGAP2AS1 into exosomes and its secretion outside breast cancer cells is done in an hnRNPA2B1-dependent manner (Zheng et al., 2019). Also, Gao et al. showcased the contribution of hnRNPK in the packaging of lncRNA 91H in colorectal cancer (CRC) which caused aggressive tumor relapse and metastasis (Gao et al., 2018). The study conducted by Liu et al. highlights the crucial role of exosomal long noncoding RNAs (lncRNAs), with a particular focus on LINC01133, in the progression of pancreatic ductal adenocarcinoma (PDAC), an extremely aggressive and lethal form of cancer (Figure 5). It was found that LINC01133 is not just highly present in PDAC cases but also associated with more advanced cancer stages and lower survival rates in patients. The study explores how LINC01133 affects PDAC progression, showing that Periostin increases both exosome release and LINC01133 levels, which in turn activates several cancer-promoting processes such as cell growth, movement, invasion, and the transition from epithelial to mesenchymal states via the EGFR pathway and interaction with c-myc. Crucially, LINC01133 boosts the Wnt/β-catenin signaling pathway through its interaction with EZH2, resulting in AXIN2 suppression and β-catenin activation via H3K27 trimethylation. These insights highlight the central role of exosomal LINC01133 in PDAC and propose that targeting LINC01133 could be an effective approach for treating this cancer (van Niel et al., 2018).

### 4.1 The selective sorting mechanism of lncRNAs into exosomes

The type of a cell and its homeostatic state define the ncRNAs that load into an exosome with other cargos; however, not much is known about the exact mechanisms and factors that contribute to the selective sorting of lncRNAs into exosomes (Qiu et al., 2021). Nevertheless, some nucleotide sequences have been identified on RNAs that can determine how RNAs are chosen and then packed into exosomes (Groot and Lee, 2020; Hoek et al., 1998; Hwang et al., 2007; Wang et al., 2010b). Ahadi et al., investigated some exosomal lncRNAs secreted from four different prostate cancer cell lines (VCaP, LNCaP, DU145, PC3) and one normal prostate cell line. The aim was to determine if any specific RBP binding sites on lncRNAs could be found. They were able to identify 126 different six‐base motifs that were particularly expressed in the four prostate cancer cell lines but not the healthy prostate cell line (Ahadi et al., 2016). Chen et al. found that there is a sequence of GGAG on the 1930-1960 nt region of LNMAT2 that binds with hnRNPA2B1 specifically. This sequence is located on a stem-loop structure and is recognized by hnRNPA2B1 (Chen et al., 2020a). In the case of exosomal lncRNA H19 originated from NSCLC, Lei et al. found the same special sequence of GGAG at the 5′ -end of the lncRNA which was the specific binding site of hnRNPA2B1 (Lei et al., 2018). Moreover, in RCC cells, lncARSR could bind to hnRNPA2B1 through its special motifs (GGAG/CCCU) at the 5′ terminal region (Qu et al., 2016).

### 4.2 Role of exosomal lncRNAs in tumor microenvironment modulation

A tumor microenvironment (TME) is a mixture of non-cancerous cells, blood vessels, secreted factors, and extracellular matrix (ECM), which collectively promote the growth of a tumor. From the beginning of the tumor growth, the cancer cells start to have interactions with their surrounding normal cells through secreting exosomes and will induce the transformation of their local environment to shape the desired TME that supports immunosuppression, cancer cell survival, local invasion, and metastatic dissemination (Chen et al., 2019a; Jin and Jin, 2020; Pathania and Challagundla, 2021). LncRNAs constitute a significant portion of this cell-cell communication between cancer cells and TME (Pathania and Challagundla, 2021). LncRNAs secreted from tumor cells can affect ECM, stromal cells, immune cells, endothelial cells, macrophages, and myeloid-derived suppressor cells (MDSCs) (Chen et al., 2019a). One major event during tumor progression is the lack of oxygen supply or hypoxia. This situation leads to the activation of hypoxia-inducible factor (HIF)-1α pathway and eventually a great deal of lncRNAs are produced and secreted into TME that elevate cell survival (Masoud and Li, 2015; Takahashi et al., 2014b). For instance, in BC cell line 5637, lncRNA urothelial cancer-associated 1 (UCA1) is secreted during hypoxia condition and aids tumor promotion via epithelial-mesenchymal transition (EMT) (Xue et al., 2017). In another study it was shown that CCAL (colorectal cancer-associated lncRNA) is transferred from cancer-associated fibroblasts (CAFs) to cancer cells via exosomes. Once inside the CRC cells, CCAL suppresses apoptosis, enhances chemoresistance, and activates the β-catenin pathway, both *in vitro* and *in vivo* (Deng et al., 2020). Furthermore, Liang et al., found that exosomes derived from CRC cells transport lncRNA RPPH1 into macrophages. This transfer promotes the polarization of macrophages into the M2 phenotype, which in turn facilitates metastasis and proliferation of CRC cells. Additionally, levels of exosomal RPPH1 in blood plasma were higher in treatment-naive CRC patients but decreased after tumor resection (Liang et al., 2019). Another study on CRC showed that the expression of lncRNA 91H in serum was closely associated with exosomes, both *in vitro* and *in vivo*. The association was likely to enhance tumor-cell migration and invasion during tumor development by altering HNRNPK expression. Also, CRC patients with high levels of lncRNA 91H expression had a higher risk of tumor recurrence and metastasis compared to patients with low lncRNA 91H expression (Gao et al., 2018). Moreover, Pan et al., found that the expression of ZFAS1 was elevated in tumor tissues, serum, and serum exosomes of GC patients, which was significantly correlated with lymphatic metastasis and the TNM stage of the disease (Pan et al., 2017).

Some other exosomal lncRNAs have been identified that can contribute to cell growth (Zhao et al., 2019a), proliferation (Zhao et al., 2019a; Xu et al., 2020a; Hu and Hu, 2019; Conigliaro et al., 2015), angiogenesis (Chen et al., 2020a; Conigliaro et al., 2015), migration (Chen et al., 2020a; Zhao et al., 2019a; Xu et al., 2020a; Hu and Hu, 2019; Chen et al., 2020b; Feng et al., 2019), invasion (Xu et al., 2020a; Chen et al., 2020b; Lu et al., 2020), metastasis (Chen et al., 2020a; Zhao et al., 2019a), drug resistance through activation of Wnt/β-catenin pathway in recipient cells (Deng et al., 2020), inhibition of apoptosis (Chen et al., 2020b; Yin et al., 2020; Song et al., 2020), and inhibition of inflammation (Song et al., 2020; Li et al., 2018a), all of which ultimately promotes tumor growth. Lastly, recent studies have pointed out the involvement of exosomal lncRNAs in autophagy. Tumor cells tend to upregulate autophagy in order to minimize environmental stress, protect themselves from chemotherapy, and maintain their fuel supply. Exosomal lncRNAs have been proven to play a significant role in inducing autophagy which leads to cell survival and proliferation (Pathania and Challagundla, 2021; Sun et al., 2013). For example, high lncRNA-CAF levels in normal stromal fibroblasts reinforce tumor proliferation, and are associated with poor prognosis in oral squamous cell carcinoma (OSCC) patients (Ding et al., 2018). Table 3 details the specific roles of long non-coding RNAs (lncRNAs) contained within exosomes in the context of tumor microenvironment modulation.

**TABLE 3:** Role of Exosomal lncRNAs in Tumor Microenvironment Modulation.

Exosomal lncRNA	Model systems	Source cell type	Target cell type	Modulatory effect	Mechanism of action	References
MALAT1	*In silico*/In vitro (tissue samples, blood samples, and cell lines)/In vivo (female BALB/c nude mice)	Triple-negative breast cancer (TNBC) cells	Macrophages	Induces M2 polarization	Activated the Hippo/YAP axis by up-regulating POSTN	Wang et al. (2024a)
HOTAIR	In vitro (tissue samples and cell lines)/In vivo (male BALB/c nude mice)	Laryngeal squamous cell carcinoma (LSCC)	Macrophages	Induces M2 polarization	Activates the PI3K/p-AKT/AKT pathway and promotes EMT and metastasis	Wang et al. (2022)
GAS5	*In silico*/In vitro (tissue samples and cell lines)/In vivo (female BALB/c nude mice)	Non-small cell lung cancer (NSCLC) cells	T cells	Suppresses anti-tumor immune response	Binds to and inhibits the activity of the tumor suppressor p53	Wang et al. (2024b)
H19	In vitro (tissue samples and cell lines)/In vivo (NOD/SCID mice)	Cancer-associated fibroblasts (CAFs)	Colorectal Cancer cells	Promotes proliferation and metastasis	Activates Wnt/β-catenin signaling pathway by acting as a ceRNA for miR-141	Ren et al. (2018)
lncRNA-p21	In vitro (tissue samples, blood samples, and cell lines)	NSCLC cells	Endothelial cells	Promotes angiogenesis and tumor cell adhesion	Increases the expression of microRNAs (miR-23a, miR-146b, miR-330, and miR-494) and genes (GLUT1, PFKFB3) related to endothelial cell activation	Castellano et al. (2020)
SOX2-OT	*In vitro* (THP-1, NSCLC, and H1975 cells)	NSCLC cells	Macrophages	Induces M2 polarization	Targets miR-627-3p/SMADs pathway	Zhou et al. (2021b)
PCAT6	*In vitro* (BEAS-2B and NSCLC cells)	NSCLC cells	Macrophages	Induces M2 polarization	Increases migration, invasion and EMT processes through the miR-326/KLF1 axis	Chen et al. (2022)
LINC00313	*In vitro* (cell lines)/In vivo (mouse-xenograft models)	NSCLC cells	Macrophages	Induces M2 polarization	Activates STAT6 signaling pathway	Kong et al. (2022)
MEG3	In vitro (tissue samples and cell lines)/In vivo (BALB/c nude mice)	Cancer-associated fibroblasts (CAFs)	small-cell lung cancer (SCLC)	promotes chemoresistance, cell viability and metastasis	Promotes cancer progression by regulating the miR-15a-5p/CCNE1 axis	Sun et al. (2022)
TUG1	In vitro (tissue samples and cell lines)	Cancer-associated fibroblasts (CAFs)	Hepatocellular carcinoma (HCC) cells	Promotes migration, invasion, and glycolysis	Interacts with miR-524-5p, which targets SIX1	Lu et al. (2022)
SNHG12	*In silico*/In vitro (cell lines)/In vivo (female athymic BALB/c nude mice)	Breast cancer cells	Endothelial cells	Enhances angiogenesis	Promoting the advancement of breast cancer by interacting with PBRM1 to influence the activity of MMP10	Chen et al. (2024)
SNHG14	In vitro (tissue samples, blood samples, and cell lines)	Breast cancer cells	Normal fibroblasts (NFs)	Facilitates cell viability and migration	Induces the transformation of NFs into CAFs by the EBF1/FAM171A1 axis	Dong et al. (2023)
AFAP1-AS1	In vitro (cell lines)/In vivo (male NOD-SCID mice)	Pituitary adenomas (PA) cells	Cancer cells	Enhances PA cell growth, metastasis, and glucose metabolism	Inhibits the SMURF1-mediated ubiquitination of HuR, leading to increased expression of HK2 and PKM2	Tang et al. (2024)
CRNDE-h	In vitro (tissue samples, blood samples, and cell lines)/In vivo (male C57BL/6 mice)	Colorectal cancer cells	T helper 17 (Th17) cells	Promotes cell differentiation	Binds to the PPXY motif of RORγt and inhibits its ubiquitination	Sun et al. (2021a)
UCA1	In vitro (serum samples and cell lines)/In vivo (female nude mice)	Pancreatic cancer (PC)	Endothelial cells	Promotes angiogenesis and tumor growth	Promotes cancer progression by targeting the miR-96-5p/AMOTL2/ERK1/2 axis	Guo et al. (2020b)
HOTTIP	In vitro (tissue samples, blood samples, and cell lines)	Mitomycin-resistant colorectal cancer cells (CRC)	Mitomycin-sensitive colorectal cancer cells (CRC)	Increases the resistance of colorectal cancer cells to mitomycin	Decreases the expression of miR-214, which leads to an increase in KPNA3 expression	Chen et al. (2020c)
XIST	*In silico*/In vitro (CT26, HCT116, and NCM460 cells)	Colon cancer cells	Macrophages	Induces M2 polarization	Serves as a sponge for miR-17-5p, resulting in the upregulation of PDGFRA and promoting tumorigenesis	Gao et al. (2024)
linc-ROR	*In vitro* (serum samples and cell lines)/In vivo (male BALB/c nude mice)	Pancreatic cancer (PC)	Adipocytes	Induces dedifferentiation of adipocytes into preadipocyte/fibroblast-like cells and facilitates EMT	Induces PC cell EMT via the HIF1α-ZEB1 axis	Sun et al. (2021b)
LINC00161	*In vitro* (tissue samples, blood samples, and cell lines)/In vivo (female BALB/c nude mice)	Hepatocellular carcinoma (HCC)	Endothelial cells	Enhances angiogenesis	Inhibits miR-590-3p to activate the ROCK2 signaling pathway	You et al. (2021)
ZFAS1	*In vitro* (cell lines)/In vivo (female BALB/c nude mice)	Esophageal squamous cell carcinoma (ESCC)	Cancer cells	Promotes cell proliferation, invasion and migration	Competitively regulates miR-124, leading to STAT3 activation	Li et al. (2019b)
	*In vitro* (tissue samples and cell lines)	Gastric cancer (GC)	Cancer cells	Promotes proliferation, migration and lymphatic metastasis	Promotes the upregulation of cyclin D1, Bcl2, N-cadherin, Slug, Snail, Twist, and ZEB1while downregulating f Bax and E-cadherin	Pan et al. (2017)
RPPH1	*In vitro* (tissue samples and cell lines)/In vivo (male BALB/c nude mice)	Colorectal cancer cells (CRC)	Macrophages	Induces M2 polarization, promotes metastasis and proliferation	Interacts with β-III tubulin (TUBB3) to prevent its ubiquitination	Liang et al. (2019)

## 5 Drug resistance in gynecological cancers

### 5.1 Mechanisms underlying drug resistance in cancer cells

Drug resistance has long been an issue in many cancer studies. Despite the efforts and successes in cancer treatment, anticancer drug resistance remains a major problem in the treatment of this disease and has a significant impact on the clinical outcome (Mondal and Meeran, 2021; Vasan et al., 2019). In general, there are two types of drug resistance: intrinsic, which exists before treatment starts, and acquired resistance. In acquired resistance, cancer cells that have already received the drugs can acquire resistance to the treatment at later stages, which is the most important cause of relapse and death in cancer patients (Wang et al., 2019a). Chemical resistance can be divided into two categories: single-drug resistance and multidrug resistance. In the case of single-drug resistance, the cancer cells are resistant to a specific drug. In contrast, multidrug resistance occurs when the cancer cells are resistant not only to the drugs currently being used, but also to other chemotherapeutic agents to which they have not previously been exposed (Gacche and Assaraf, 2018). MDR is one of the most important mechanisms in the development of drug resistance and is responsible for more than 90% of deaths in cancer patients receiving anticancer drugs. The major mechanisms in the development of MDR include overexpression of ABC transporters, defects in the apoptotic system, alterations in drug metabolism or drug targets, epigenetic changes, oncogene amplification, and increased DNA repair capacity (Bukowski et al., 2020; Yang et al., 2014; Vaidya et al., 2022).

### 5.2 Specific drugs and their resistance profiles in gynecological cancers

#### 5.2.1 Breast cancer

Currently, most breast cancer therapies are based on targeted treatments, as this disease has considerable heterogeneity. ER+ (estrogen receptor) and PR+ (progesterone receptor) breast cancers belong to the subtype of hormone-dependent cancers (Kinnel et al., 2023). Approximately 70%–80% of all breast cancers are HR+, which can be effectively treated by endocrine therapy or anti-estrogen therapy by modulating ER or lowering estrogen levels. One of the main drugs in the treatment of HR + breast cancer is tamoxifen. This drug is a selective estrogen receptor modulator that blocks the effect of estrogen on ER-positive breast cancer cells. Hormone therapy for premenopausal women includes administration of tamoxifen alone or a combination of a luteinizing hormone-releasing hormone analog with tamoxifen or an aromatase inhibitor (AI). Postmenopausal women are treated with aromatase inhibitors such as letrozole and exemestane or tamoxifen combined with SERDs (selective estrogen receptor degraders) like fulvestrant (Kay et al., 2021). Also, abemaciclib is an inhibitor of cyclin-dependent kinase 4 and 6 (CDK4/6) that was recently approved for HR + advanced breast cancer in combination with endocrine therapy (Johnston et al., 2020). The role of tyrosine kinase receptors (activation of the PI3K/AKT/mTOR pathway), transcription factors (activation of the C-MYC/HDAC5/SOX9 axis), cell cycle regulators (interaction of LEM4 with CDK 4/6 and Rb to accelerate the G1–S transition), and autophagy (activation of LAMP3) are among the mechanisms involved in tamoxifen resistance (Yao et al., 2020). 15%–20% of breast cancers have overexpression of the HER-2 gene and are more aggressive than HER-2 negative breast cancers (Le Du et al., 2021). Trastuzumab, a recombinant humanized monoclonal antibody, has been one of the first drugs used in the treatment of HER-2+ breast cancer. First-line therapy for most patients consists of a mix of trastuzumab, pertuzumab (another monoclonal antibody), and taxanes (a class of chemotherapeutic agents), while trastuzumab emtansine (T-DM1) is the second-line therapy (Kay et al., 2021; Le Du et al., 2021; Martínez-Sáez and Prat, 2021). Several novel treatments such as tucatinib, lapatinib, neratinib, fam-trastuzumab deruxtecan-nxki (DS-8201a), and margetuximab-cmkb that have recently been approved are among the third-line treatment options (Martínez-Sáez and Prat, 2021). Despite medical advances in the treatment of this type of breast cancer, approximately 22%–25% of HER-2-positive metastatic breast cancer patients have congenital or acquired drug resistance, which is associated with significant morbidity and mortality (Choong et al., 2020). Mechanisms of drug resistance in HER-2+ breast cancer include inadequate blockade of the HER-2 receptor, activation of downstream signaling pathways like PI3K and MAPK, inhibition of tumor suppressor genes, acquired HER-2 mutations, dysregulation of cell cycle regulators (high copy number of the CCND1 gene encoding cyclin D1), and non-cell autonomous mechanisms within the tumor microenvironment (Kay et al., 2021; Choong et al., 2020; Zhang, 2021). Triple negative breast cancer (TNBC) lacks ER, PR, and HER-2 receptors. It accounts for 15%–20% of all breast cancers, and due to its heterogeneity and complexity, treatment strategies are usually unsuccessful (Merikhian et al., 2021; Marra et al., 2020). Currently, chemotherapy is the first line of treatment, and most patients become resistant to chemotherapeutic agents (Cosentino et al., 2021). Conventional chemotherapy used in TNBC treatment can be divided into two categories: neoadjuvant therapy and adjuvant therapy. Anthracycline–cyclophosphamide drugs (AC regimen) such as doxorubicin, cyclophosphamide and paclitaxel are used in neoadjuvant therapy, which can improve the efficacy of treatment when combined with cisplatin. Other neoadjuvant drugs include carboplatin, Abraxane, and bevacizumab (a monoclonal antibody that inhibits VEGF activation). Neoadjuvant therapy also utilizes anthracycline-taxane like doxorubicin, docetaxel, and cyclophosphamide. In cases of advanced or metastatic breast cancers where these drugs are ineffective, capecitabine may be given alone or in combination with docetaxel (Medina et al., 2020). Some mechanisms involved in TNBC chemoresistance are upregulation of ABC transporters (an important mechanism), presence of cancer stem cells (CSCs), hypoxia, and TP53 mutations (Marra et al., 2020).

#### 5.2.2 Ovarian cancer

Ovarian cancer treatment generally begins with surgical removal and is followed by chemotherapy, radiotherapy, or neoadjuvant chemotherapy (Alatise et al., 2022). High-dose chemotherapy usually leads to drug resistance development in patients, and about 80% of patients experience a relapse after treatment and gradually become resistant to chemotherapeutic agents (Alatise et al., 2022; Brasseur et al., 2017). Platinum-based and taxane-based drugs, including cisplatin (DDP), are mainly used to treat ovarian cancer, and resistance to these drugs has caused many problems so far (Alatise et al., 2022; Cui, 2022; Zhang et al., 2023). In advanced cases, a combination of cisplatin with gemcitabine is recommended to increase the response to cisplatin treatment (Garrido et al., 2021).

Ovarian cancer (OC) is categorized into two main types: epithelial OC and non-epithelial OC. Epithelial ovarian cancer (EOC) is the most common type, accounting for about 90% of all malignant ovarian tumors. EOC consists of several subtypes, including endometrioid, clear cell, serous carcinoma [low-grade serous carcinoma (LGSOC) and high-grade serous ovarian carcinoma (HGSOC)], undifferentiated, and mucinous. Among these, HGSOC is the most common subtype and is responsible for 75% of EOC cases (Lukanović et al., 2022; Gaona-Luviano et al., 2020; Shih et al., 2021). Recently, polyadenosine diphosphate ribose polymerases (PARP) inhibitors (niraparib, cediranib, olaparib, and pembrolizumab) and anti-angiogenic options (trebananib, sorafenib, entrectinib) have been shown to be effective in the treatment of epithelial origin (EOC) (Garrido et al., 2021). The endometrioid and clear cell subtypes make up 20%–25% and 5%–10% of EOCs, respectively (Zhou et al., 2021c). In one study, a signaling pathway was discovered that can be targeted to increase the sensitivity of platinum-resistant ovarian endometrioid cancer cells to chemotherapy. The study revealed that using non-receptor tyrosine kinase Lymphocyte Cell-Specific Protein-Tyrosine Kinase inhibitors (LCKi) followed by co-treatment with cisplatin resulted in lower cell viability and increased cell death in laboratory tests. This effect was associated with increased DNA adduct formation and reduced tumor growth *in vivo* (Crean-Tate et al., 2021). Clear cell carcinoma (CCC) is one of the most common chemoresistant cancers. However, the precise mechanisms underlying drug resistance in this type of cancer are not fully understood. Studies have demonstrated that genes associated with the epithelial-mesenchymal transition (EMT) pathway are markedly elevated in cases of ovarian CCC that do not respond to chemotherapy. This suggests that the EMT pathway plays a crucial role in the development of chemotherapy resistance (Takahashi et al., 2023). Although 60%–80% of patients with HGSOC initially exhibit a positive response to treatment, most will eventually develop resistance to platinum-based therapies. Epigenetic modifications like DNA methylation, histone deacetylation, and microRNA expression have been linked to the development of chemoresistance in HGSOC. For instance, hypomethylation of MSX1 contributes to EMT by promoting cancer cell transition to a mesenchymal phenotype, resulting in chemoresistant HGSOC patients. These modifications could be targeted by epigenetic modulation therapies to overcome chemoresistance (Matthews et al., 2021).

Non-epithelial ovarian cancers (NEOC) are rare malignancies, accounting for approximately 10% of all ovarian cancer cases. This group mainly comprises germ cell tumors (GCT), sex cord-stromal tumors (SCST), and a few exceptionally rare tumor subtypes (Martínez-Sáez and Prat, 2021). GCTs and SCSTs represent 2%–5% and 2% of ovarian cancers, respectively. Cisplatin-based chemotherapy with surgery can effectively treat most GCTs. However, resistance to cisplatin (CDDP) can develop due to changes in the levels of critical factors such as p53, mouse double minute 2 homolog (MDM2), octamer-binding transcription factor 4 (Oct4), and cytoplasmic p21. Due to the lack of high-quality studies in this area, current practice guidelines recommend using chemotherapy regimens established for testicular germ cell tumors due to their similar origin (De Giorgi et al., 2019; Uccello et al., 2020). The current targeted therapy approaches being studied in clinical trials for GCTs and SCSTs include tyrosine kinase inhibitors, angiogenesis inhibition, immunotherapy, and endocrine therapy. However, there is still limited research on many aspects of non-epithelial ovarian tumors, especially their resistance to chemotherapy (Maoz et al., 2020).

Examples of chemotherapy resistance mechanisms in ovarian cancer include expression of anti-apoptotic proteins, overexpression of MAPK-activating death domain protein (MADD), disruption of major DNA repair pathways, increased efflux due to high expression of APC transporters, and the presence of a small subset of cancer stem cells (CSCs) (Alatise et al., 2022; Ortiz et al., 2022).

#### 5.2.3 Cervical cancer

Cervical cancer has two main types: squamous cell carcinoma (SCC), accounting for about 75% of cases, and adenocarcinomas, responsible for approximately 25% of cervical cancers.

High-risk human papillomavirus (HPV) infection, predominantly types 16 and 18, is considered a major factor in the development of most SCCs (Volkova et al., 2021; Liu et al., 2023). Although, HPV-independent tumors are associated with both adenocarcinomas and squamous histologic subtypes (Fernandes et al., 2022). HPV-negative cervical cancers have a more aggressive course, which is clinically significant (Volkova et al., 2021). HPV oncoproteins like E6 and E7, which inactivate tumor suppressor genes p53 and Rb, further promote resistance by allowing cells to evade apoptosis (Xiong et al., 2022).

Surgery and radiotherapy are the main treatments of cervical cancer, and chemotherapy is often used if metastasis or tumor recurrence happens (Li et al., 2017). One of the main chemotherapeutic agents used to treat cervical cancer is cisplatin, which exerts its effects by attacking DNA and forming irreparable bonds with it (Mahapatra et al., 2022). Research has shown that the combination of platinum and paclitaxel has better efficacy in treatment response compared with cisplatin alone (Della Corte et al., 2020). In advanced cervical cancer, patients become resistant to these drugs, and cancer cells evade its anti-cancer effects; therefore, finding a solution to overcome the acquired resistance is of great importance (Mahapatra et al., 2022; Liu et al., 2016a). Acquired cisplatin resistance in cervical cancer therapy is due to decreased uptake of cisplatin (downregulation of the transmembrane protein CTR1), increased efflux (overexpression of MRP1), inactivation of the apoptotic pathway, inactivation of proteins containing thiol groups, and activation of the epithelial-mesenchymal transition (EMT) (Bhattacharjee et al., 2022).

#### 5.2.4 Endometrial cancer

Endometrial cancer is a common gynecological cancer that affects the uterus. According to Bokhman’s classification, it is divided into two types: estrogen-independent, which has a poorer outlook and is often diagnosed late, and estrogen-dependent endometrial cancers. Moreover, WHO classifies endometrial cancer into four types: endometrioid endometrial cancer (EEC), serous adenocarcinoma, clear cell adenocarcinoma and mixed endometrial cancer and uterine carcinosarcoma. EECs are generally estrogen-dependent tumors and are the most common type of endometrial cancer, accounting for more than 80% of cases (Yang et al., 2024). Treatment typically involves surgery, vaginal brachytherapy, external beam radiotherapy (EBRT) and in advanced cases, carboplatin, paclitaxel, adriamycin, and cisplatin may be administered (Saito et al., 2021). For hormone receptor-positive cancers, hormone therapies such as progestins, gonadotropin-releasing hormone agonists, or aromatase inhibitors are used (Kailasam and Langstraat, 2022). Emerging treatments include immune checkpoint inhibitors like pembrolizumab, particularly for cancers with microsatellite instability (MSI) or mismatch repair deficiency (dMMR), and targeted therapies such as PI3K/AKT/mTOR inhibitors (Yang et al., 2024; Jeong et al., 2023; Venkata et al., 2022; Colombo et al., 2024). Resistance to treatment in endometrial cancer often occurs due to activation of the PI3K/AKT/mTOR pathway, especially in tumors with mutations in the PIK3CA gene or loss of PTEN function. These mutations promote cell survival and reduce the effectiveness of hormonal and chemotherapeutic treatments (Yang et al., 2024; Lee, 2021). Additionally, some endometrial cancers that initially respond to immunotherapy can develop resistance by upregulating immune evasion mechanisms, such as increased PD-L1 expression, while hormone therapy resistance is often linked to mutations in estrogen and progesterone receptors (Yang et al., 2024; Hao et al., 2024). Table 4 provides a list of various drugs and how they cause drug resistance in different types of cancers.

**TABLE 4:** Specific drugs and their resistance profiles in gynecological cancers.

Drug name	Cancer type	Mechanism of action	Common resistance mechanisms	Clinical impact of resistance	Strategies to overcome resistance	References
Cisplatin	Ovarian, Cervical, Endometrial	DNA damage (platinates)	Mutations in BRCA genes, increased DNA repair	Reduced tumor response, disease progression	PARP inhibitors, combination with other chemotherapies	Kelland (2007) and Konstantinopoulos and Matulonis (2018)
Paclitaxel	Ovarian, Cervical, Endometrial	Disrupts cell division (taxanes)	Mutations in tubulin genes, altered drug efflux	Decreased tumor sensitivity, recurrence	Docetaxel, combination with other chemotherapies, targeted therapies	Weaver (2014) and van Zyl et al. (2018)
Bevacizumab	Ovarian, Cervical	Inhibits blood vessel growth (anti-angiogenic)	Tumor mutations in VEGF signaling pathway, development of alternative blood supply	Limited response to therapy, poorer prognosis	Combination with chemotherapy, targeted therapies targeting other pathways	Ortiz et al. (2022), Pujade-Lauraine et al. (2019), and Jin et al. (2022)
Tamoxifen	Hormone-receptor positive Breast & Endometrial	Blocks estrogen receptor activity (hormonal)	Mutations in estrogen receptor gene, reduced expression of receptor	Hormone therapy becomes ineffective, disease progression	Aromatase inhibitors, fulvestrant, targeted therapies against PI3K pathway	Jordan (2003), Osborne and Schiff (2011), and Mills et al. (2018)
Letrozole	Hormone-receptor positive Breast	Inhibits estrogen production (aromatase inhibitor)	CYP19A1 gene mutations, acquired resistance to tamoxifen	Limited response to tamoxifen, disease progression	Combination with other hormonal therapies, targeted therapies	Miller (2003) and Magnani et al. (2017)
Pembrolizumab	Ovarian, Cervical	Enhances immune response (immune checkpoint inhibitor)	Loss of PD-L1 expression on tumor cells, impaired immune function	Reduced tumor response, limited benefits for most patients	Combination with chemotherapy, targeted therapies, development of new immune checkpoint inhibitors	Hamanishi et al. (2015) and Santoro et al. (2024)
Niraparib	Ovarian	Inhibits PARP enzyme (PARP inhibitor)	BRCA reversion mutations, loss of DNA repair mechanisms	Disease progression, limited options for further treatment	Combination with other PARP inhibitors, clinical trials of new PARP inhibitor combinations	Mirza et al. (2016) and Lord and Ashworth (2017)
Docetaxel	Ovarian, Endometrial	Disrupts cell division (taxanes)	Similar to Paclitaxel	Reduced tumor response, recurrence	Combination with other chemotherapies, targeted therapies, platinum-based drugs	McGuire and Markman (2003) and Katsumata et al. (2009)
Pembrolizumab (combination with Bevacizumab)	Ovarian	Enhances immune response and inhibits blood vessel growth	Combination of resistance mechanisms from both drugs	Limited response to therapy, poorer prognosis	Other immune checkpoint inhibitors, targeted therapies against alternative pathways, clinical trials of novel combinations	Zamarin and Jazaeri (2016), Colombo et al. (2023), and Zsiros et al. (2021)
Lenvatinib	Endometrial	Targets multiple pathways (angiogenesis, cell proliferation)	Mutations in target genes, increased drug efflux	Disease progression, limited response to other therapies	Combination with other targeted therapies, clinical trials of new drug combinations	Makker et al. (2020) and Walker et al. (2023)

## 6 Role of LncRNAs in specific gynecological cancers

LncRNAs have emerged as significant players in the molecular landscape of various cancers, including gynecological cancers. Table 5, which focuses on clinical trials investigating the role of lncRNAs in cancers, provides an overview of the ongoing and completed studies aimed at understanding how these molecules influence cancer development, progression, and response to treatment.

**TABLE 5:** Clinical trials investigating the role of long non-coding RNAs (lncRNAs) in cancers.

Trial identifier	Cancer type	lncRNA targeted	Objective	Study design	Outcome measures
NCT05088811	Hepatocellular Carcinoma	WRAP53, UCA-1	Diagnostic value of WRAP53 and UCA-1 in hepatocellular carcinoma	Observational Model: Case-Control	Diagnostic accuracy of WRAP53 and UCA-1
NCT03000764	Breast Carcinoma	Non-coding RNAs	Transcriptomic profiling in radiation-induced fibrosis	Interventional: Single Group	Transcriptomic signature in fibroblasts and serum
NCT05708209	Oral Squamous Cell Carcinoma	MALAT1	Salivary determination of MALAT1 and miRNA-124	Observational Model: Other	Quantitative determination of MALAT1 and miRNA-124
NCT03830619	Lung Cancer	Serum exosomal lncRNAs	Expression of serum exosomal lncRNAs as biomarkers for lung cancer diagnosis	Observational Model: Cohort	Levels of exosomal lncRNAs, tumor biomarkers, and CT scan results
NCT06357689	Breast Cancer	LINC00511	Relationship of LINC00511 SNPs with breast cancer risk	Observational Model: Case-Control	SNP associations with breast cancer and hormone receptor status
NCT03469544	Thyroid Cancer	HOTAIR	Role of HOTAIR and serum midkine in thyroid cancer	Observational Model: Case-Control	Expression levels of HOTAIR and serum midkine
NCT06544005	Hepatocellular Carcinoma	HOTTIP	HOTTIP expression and its role in liver cancer metastasis	Observational Model: Case-Control	Primary outcome analysis
NCT06427720	Breast Cancer	LINC00511, miR-185-3p, miR-301a-3p	Analyze expression and interaction between miRNAs and lncRNA	Observational Model: Case-Control	Expression patterns and relationships among the markers
NCT06459895	Bronchial Asthma	MALAT1	MALAT1 gene expression and TNF-alpha as biomarkers	Observational Model: Case-Control	Gene expression levels of MALAT1 and TNF-alpha
NCT03738319	Ovarian Cancer	miRNA/lncRNA	Differential expression of miRNA/lncRNA in ovarian cancer	Observational Model: Case-Control	Expression profiles and progression-free survival
NCT06432413	Colorectal Cancer	SNHG3, LUNAR1	Measure expression levels and patterns of Notch-associated lncRNAs in relation to tumor features	Observational Model: Case-Control	Expression levels of SNHG3 and LUNAR1
NCT04269746	Colorectal Cancer	CCAT1	Diagnostic value of CCAT1 in colorectal cancer	Observational Model: Case-Crossover	CCAT1 expression levels
NCT06427278	Colorectal Cancer	CCDC144NL-AS1	Role of CCDC144NL-AS1 and HMGA2 protein in CRC	Observational Model: Case-Control	Expression patterns and clinical associations

## 7 Exosomal lncRNAs and their role in drug resistance

### 7.1 Evidence linking exosomal lncRNAs to drug resistance

Approximately 98% of the human genome is made up of ncRNAs. They were once believed to be transcriptional waste, but recent studies have proven this theory wrong. ncRNAs are in fact functional factors with the ability to regulate a great number of molecular processes (Wang et al., 2021b). It was discovered that certain lncRNAs are upregulated in cancer cells or patients, as well as in exosomes present in serum or other body fluids of patients. Further investigations proved that these exosomal lncRNAs could actually aid a number of cancer cell characteristics including drug resistance, as well as conferring these characteristics in new cells through exosomal transfer. This notion was proven when exosomal lncRNAs that showed correlation with other contributing factors in cancer drug resistance were targeted in a number of malignancies and resistant cells were resensitized to cancer drugs (De Los Santos et al., 2019; Wang et al., 2021b; Guo et al., 2020c). For example, downregulation of lncRNA HOXA transcript at the distal tip (HOTTIP) made gastric cancer (GC) cells sensitive to cisplatin. In addition, HOTTIP were packaged into exosomes and transmitted to sensitive cells, which made them resistant to cisplatin (Wang et al., 2019b). High levels of SBF2 antisense RNA 1 (SBF2-AS1) were detected in exosomes secreted from glioblastomas (GBM) cells and were associated with poor response to temozolomide (TMZ). These exosomes were also able to transfer lncRNA SBF2-AS1 to chemoresponsive cells. Therefore, the previously sensitive cells would show resistance to TMZ (Zhang et al., 2019). In another study, urothelial carcinoma-associated 1 (UCA1) levels were much higher in cetuximab-resistant colorectal cancer (CRC) cells than that of sensitive cells. The same trend was noticed in progressive disease patients compared to partially or completely responsive patients. Moreover, exosomes from resistant cells could modulate UCA1 expression in recipient sensitive cells and disseminate resistance in them (Yang et al., 2018). Takahashi et al. also investigated the role of lincRNA-ROR (linc-ROR) in hepatocellular carcinoma (HCC) cells. After confirming upregulation of linc-ROR in sorafenib-resistant cells, cells were treated with small interfering RNA (siRNA) lincRNA-ROR-1. This resulted in increased early apoptosis and total apoptotic cells. It was concluded that linc-ROR contributed to apoptosis suppression and cell survival promotion by altering chemosensitivity (Takahashi et al., 2014c).

### 7.2 Mechanistic insights: how exosomal lncRNAs confer resistance

#### 7.2.1 Cell cycle

Integral transmission of genetic information is a direct consequence of faultless DNA replication and orderly progression of cell cycle (Brandmaier et al., 2017). Principally, cell cycle is a conserved process in organisms, from unicellular eukaryotes to complex metazoans (Asghar et al., 2015). This procedure takes place in accordance with cell signals that mark the right time of phase transition (G0/G1, S, G2, and M) (Otto and Sicinski, 2017). Errors in cell cycle and the resulting uncontrolled cell proliferation is one of the fundamental characteristics of cancer (Otto and Sicinski, 2017; Malumbres and Barbacid, 2009). Cell cycle in mammals is primarily regulated by cyclin-dependent kinases (CDKs), their activators, and inhibitors. CDKs are a subfamily of regulatory enzymes that have a periodic activation and deactivation (Asghar et al., 2015; Malumbres and Barbacid, 2009; Ye et al., 2022). In cancer cells, the occurrence of genetic or epigenetic changes in the mentioned regulators leads to cell cycle disruption, which is followed by genetic instabilities such as increased DNA mutations, chromosomal abnormalities, and changes in the number of chromosomes (Malumbres and Barbacid, 2009). According to some studies, a number of exosome-derived lncRNAs have the ability to neutralize the effects of drugs that aim to repress cell proliferation and contribute to tumor progression (Ye et al., 2022).

#### 7.2.2 Apoptosis

One major mechanism of homeostasis that forms balance between cell survival and death, is apoptosis or programmed cell death. This process can be induced or inhibited by intracellular or extracellular signals through different pathways. During tumor progression, the balance between anti-apoptotic and pro-apoptotic regulators is disrupted, resulting in higher cancer cell survival (Dogan et al., 2022). Inducing apoptosis in cancer cells has been a very common approach in designing anticancer drugs, and exosomal lncRNAs seem to have developed mechanisms to interfere with drug functions. By suppressing apoptosis, lncRNAs cause drug resistance in a variety of malignancies (Takeiwa et al., 2020; Zhao et al., 2019b).

#### 7.2.3 Drug efflux

Sufficient intracellular concentration is one of the main prerequisites for anticancer drugs to exert their cytotoxic effects. In response, cancer cells have strengthened a coping mechanism named drug efflux, which can be exerted through different paths. One example is the direct transfer of drugs outside the cell by exosomes (Dong et al., 2020; Xue et al., 2022). In 2003, Shedden et al. showed that breast cancer cells are capable of expelling doxorubicin outside the cell with the help of exosomes (Shedden et al., 2003). Another way is through upregulating drug efflux pumps (Dong et al., 2020). Overexpression of the ABC transporter family is one of the most known and studied causes of multidrug resistance. ABC transporter proteins can pump chemotherapy drugs out of the cancer cell against the concentration gradient in an ATP-dependent manner, thereby reducing intracellular accumulation of the drug and protecting the cancer cell from chemotherapeutic agents. So far, 48 ABC transporters have been identified. Among the most considerable ones are multidrug resistance protein 1 (MDR1) or P-glycoprotein (P-gp), multidrug resistance-associated protein 1 (MRP1), and breast cancer resistance protein (BCRP) (International Transporter Consortium et al., 2010). Drug efflux caused by increased number of ABC transporters is one of the criteria which lncRNAs can control and cause tumor recurrence (Pathania and Challagundla, 2021).

#### 7.2.4 Autophagy

Autophagy is a regulatory self-destructive process that occurs in response to stress, such as organelle damage, the presence of abnormal proteins, hypoxia, nutrient deficiency, aging, cell death, or cancer in order to maintain homeostasis (Ariosa et al., 2021; Huang et al., 2018; Ueno et al., 2019). Currently, there are three known forms of autophagy, including macroautophagy, microautophagy, and chaperone-associated autophagy, which differ in accordance to the delivery methods of cargoes to lysosome for either degradation and recycling. The term “autophagy” usually refers to macroautophagy (Shan et al., 2021). Autophagy starts with formation of autophagosomes containing degraded components, then the autophagosome is combined with a lysosome to form an autolysosome. Finally, the internal materials are broken down and recycled (Du et al., 2021; Yun and Lee, 2018). Autophagy also plays an important role in tumor drug resistance (Chang and Zou, 2020; Li et al., 2019c). Autophagy takes two different approaches facing tumor cells. In the positive approach, autophagy causes cell death in cancer cells by suppressing apoptotic pathways. In the negative approach, on the other hand, it protects cancer cells from chemotherapy drugs. Hence, autophagy helps cancer cell survival in the time of hypoxia or metabolic stress and reduces the damages caused by antitumor drugs (Li et al., 2019c; Liu et al., 2020; New and Thomas, 2019; Sun et al., 2020). Several studies indicate the role of lncRNAs as autophagy regulators in cancer drug resistance (Bermúdez et al., 2019; Jin et al., 2020).

#### 7.2.5 Epigenetic changes

Epigenetic changes are common in tumorigenesis, and so far their role has been observed in many molecular mechanisms of cancer, such as resistance to apoptosis, DNA repair, increased drug efflux and drug detoxification (Adhikari et al., 2022). In the course of tumorigenesis, a complex network of signaling pathways are vulnerable to aberrant epigenetic changes (Toh et al., 2017). Many studies conducted in the area of drug resistance indicate the occurrence of epigenetic changes, and state that acquired drug resistance in cancer does not necessarily require a permanent genetic change (Adhikari et al., 2022; Sharma et al., 2010). Based on the studies, cancer cells apply their epigenetic modulations through methylation, acetylation or other modifications of histone and non-histone proteins (Adhikari et al., 2022).

### 7.3 Key lncRNAs implicated in resistance and their downstream targets

Studies conducted in the field of cancer drug resistance have identified a number of key exosome-derived lncRNAs playing significant roles in this regard. Deng et al. investigated the role of lncRNA colorectal cancer-associated (CCAL) in CRC cells. Higher expression levels of CCAL were recorded in the tumor stroma compared to cancer cells. Exosomal CCAL was produced in CAFs and later transferred to CRC cells, where they caused apoptosis suppression and resistance to oxaliplatin. This action was done through direct interaction of CCAL with HuR, which activates the β-catenin pathway and confers chemoresistance (Deng et al., 2020). In another study done by Ren et al., lncRNA H19 secreted by CAFs was found to be promoting chemoresistance and stemness in CRC cells. H19 would act as a ceRNA for miR-141, which is responsible for inhibiting the stemness of cells. This whole situation would first activate the β-catenin pathway and second promote the stemness of the cells (Ren et al., 2018). In a 2018 study by Yang et al., high levels of lncRNA UCA1 were found in CRC patient’s serum. Further investigations showed that UCA1 plays a role in conferring resistance to cetuximab, and if transferred to sensitive cells by exosomes, has the ability to make them resistant to the drug (Yang et al., 2018).

lncRNA HOTTIP was found to be upregulated in GC cells resistant to cisplatin. In GC cells, miR-218 was proved to bind to HOTTIP in order to inhibit its actions. In response to that, HOTTIP would activate HMGA1, a target for miR-218. By modulating miR-218/HMGA1 axis, lncRNA HOTTIP could promote cisplatin resistance in GC cells (Wang et al., 2019b). Overexpression of lncRNA SBF2-AS1 in TMZ-resistant GBM cells and tissues was observed by Zhang et al. It was discovered that ZEB1, a transcription factor, binds to SBF2-AS1 promoter region and upregulates its expression. X-ray repair cross complementing 4 (XRCC4) is an enhancer of DNA double strand breaks (DSBs) repair, and its function is limited by miR-151a-3p. In GBM cells, SBF2-AS1 acts as a ceRNA for miR-151a-3p. This eventually leads to poor response to TMZ treatment (Zhang et al., 2019).

In human HCC, the most common type of primary liver cancer, linc-ROR, a stress-responsive lncRNA, was remarkably upregulated and enriched within extracellular vesicles secreted from tumor cells. Among the liver cells, stem cells with CD133 surface markers were seen to exhibit higher rates of drug resistance. It was revealed that in CD133+ cells, transforming growth factor β (TGFβ) modulated the expression and secretion pattern of lincRNA-ROR in response to being treated with sorafenib or doxorubicin. linc-ROR was proved to participate in increasing drug resistance by modulating caspase-3/7 activity; thus contributing to a chemoresistant phenotype (Takahashi et al., 2014c).

## 8 Case studies: exosomal lncRNAs involved in drug resistance of specific gynecological cancers

Several exosome-derived lncRNAs have been identified to play crucial roles in conferring drug resistance through various mechanisms such as modulating cell cycle, apoptosis, drug efflux, autophagy, and epigenetic changes. The following sections will discuss specific case studies that illustrate how exosomal lncRNAs influence drug resistance in breast, ovarian, and cervical cancers. These studies provide insights into the molecular pathways and potential therapeutic targets that could be leveraged to overcome drug resistance in these malignancies.

### 8.1 Breast cancer: key exosomal lncRNAs and their roles in resistance

In a study conducted by Zhou et al., increased expression of lncRNA NEAT1 was observed in serum EVs of a group of breast cancer patients. The EVs were about 40–100 nm in diameter (Zhou et al., 2021d), which corresponds to the approximate diameter range of exosomes (Hao et al., 2022; Wang et al., 2016). Two breast cancer cell lines were treated with extracted EVs of the patients. The EVs were absorbed by the cell lines. Further research revealed that lncRNA NEAT1 binds to miR-141-3p, and causes KLF12 overexpression (Zhou et al., 2021d). KLF12 is a transcription factor and its overexpression has been confirmed in a number of cancers (Zhou et al., 2021d; Yang et al., 2022). Elevated expression of lncRNA NEAT1 was associated with increased proliferation, invasion, and migration, as well as resistance to cisplatin, paclitaxel, and 5-FU. Also, treating the cell lines with NEAT1 siRNA, resensitized them to the drugs, prevented colony formation, induced apoptosis, and reduced MDR1 expression (Zhou et al., 2021d). Tang et al. found that high expression of lncRNA HOTAIR-containing exosomes in serum extracted from breast tumor tissues was associated with poor response to neoadjuvant chemotherapy. It was also shown that overexpressed lncRNA HOTAIR is responsible for resistance to tamoxifen hormone therapy. To date, no information has been reported about how lncRNA HOTAIR exerts its involvement in breast cancer chemoresistance (Tang et al., 2019a). However, one study confirmed that lncRNA HOTAIR induces tamoxifen resistance in breast cancer patients through enhancing ER signaling (Xue et al., 2016). Xu et al. confirmed that the expression of lncRNA UCA1 in a tamoxifen-resistant breast cancer cell line was 20-fold higher than a sensitive cell line. Further testing showed that exosomes secreted by resistant cells can induce resistance to tamoxifen, resulting in increased cell survival and repressed apoptosis in sensitive cells. Moreover, knockdown of exosomal lncRNA UCA1 reversed the acquired resistance (Xu et al., 2016).

In a survey done by Chen et al., exosome-derived lncRNA HISLA released by TAMs in the tumor microenvironment was found to increase glycolysis in recipient breast cancer cells, which protected them from apoptosis. LncRNA HISLA binds to PHD2, which is responsible for hydroxylation of HIF-1α, then aerobic glycolysis is induced. That causes resistance to apoptosis in breast cancer cells. Interestingly, increased aerobic glycolysis results in lactate secretion into the tumor microenvironment. Lactate then increases HISLA expression via the ERK-ELK1 signaling pathway (Chen et al., 2019b). LncRNA H19 showed overexpression in doxorubicin-resistant cell lines and their secreted exosomes in a survey conducted by Wang et al. Silencing H19 caused a decline in colony forming ability and increased doxorubicin-induced apoptosis. Further, exosomes extracted from resistant cells led to a higher IC50 of doxorubicin in sensitive cells along with apoptosis inhibition and enhanced colony formation. Also, doxorubicin-resistant patients were found to have elevated serum levels of exosomal H19, which can be used as a biomarker (Wang et al., 2020). In another study lncRNA AGAP2-AS1 showed upregulation in two breast cancer cell lines with acquired trastuzumab resistance. Zheng et al. observed that knockdown of AGAP2-AS1 resensitized the cell lines to trastuzumab, indicating its involvement in the acquired resistance. The knockdown led to DNA fragmentation and apoptosis. Further results confirmed that AGAP2-AS1 is packaged into exosomes by hnRNPA2B1 and this way transfers trastuzumab resistance to sensitive breast cancer cells (Zheng et al., 2019). In a 2021 study, exosomal lncRNA AGAP2-AS1 was able to modulate autophagy through Autophagy related 10 (ATG10), which made breast cancer cells resistant to trastuzumab. AGAP2-AS1 binds to ELAV-like RNA binding protein 1 (ELAVL1/HuR), then the complex interlocks with the ATG10 promoter region and activates ATG10 transcription by causing epigenetic changes at H3K27ac (acetylation of lysine 27 on histone H3 protein subunit) and H3K4me3 (Tri-methylation of histone H3 lysine 4). ATG10 is an autophagic E2-like enzyme that aids autophagosome formation and its overexpression has been observed in cancers like colorectal and lung (Qian et al., 2021; Xie et al., 2016; Jo et al., 2012). It was also confirmed that targeting AGAP2-AS1 by Antisense oligonucleotide (ASO), reverses drug resistance (Qian et al., 2021).

### 8.2 Ovarian cancer: the lncRNA-exosome interface

Li et al. observed that lncRNA UCA1 was able to make ovarian cancer cells resistant to cisplatin. Silencing UCA1 inhibited proliferation and increased apoptosis induced by cisplatin. On the contrary, overexpression of UCA1 in sensitive cell lines inhibited cisplatin-induced apoptosis and increased tumor growth. LncRNA UCA1 targets miR-143, which is responsible for inhibiting the expression of a transcription factor called Fos-like antigen 2/FRA-2 (FOSL2). This makes ovarian cancer cells resistant to cisplatin (Li et al., 2019d; Wan et al., 2021).

### 8.3 Cervical cancer: exosomal lncRNAs as emerging evidence in cancer drug resistance

Lou et al. confirmed the involvement of lncRNA HNF1A-AS1 in triggering drug resistance in cervical cancer. LncRNA HNF1A-AS1 was upregulated in a cisplatin-resistant cell line compared to the sensitive one, and silencing it resulted in inhibited cell proliferation and increased apoptosis. Further investigation indicated that lncRNA HNF1A-AS1 acts as a ceRNA for miR-34b and inhibits its activity which is followed by Tuftelin 1 (TUFT1) upregulation (Luo et al., 2019). Overexpressed TUFT1 has been observed in a number of malignancies including hepatocellular carcinoma (HCC), breast cancer, thyroid carcinoma, and osteosarcoma (Lin et al., 2021). Table 6 illustrates the role of exosomal lncRNAs in the development of drug resistance in breast, ovarian, and cervical cancers.

**TABLE 6:** Exosomal lncRNAs involved in the drug resistance of breast, ovarian, and cervical cancers.

Type of cancer	lncRNA	Model systems	Drug	Mechanism	References
Breast	NEAT1	In vitro (serum samples, tumor tissue, MCF-7 and MDA-MB-231 cells)	Cisplatin, paclitaxel, 5-Fluorouracil	Leads to an increase in KLF by inhibiting miR-141, increasing the ability of cell proliferation, invasion, and migration	Zhou et al. (2021d)
	HOTAIR	*In vitro* (serum samples, tumor tissues, MDA-MB-231 and MCF-7 cells)	Tamoxifen, neoadjuvant chemotherapy	Not stated	Tang et al. (2019a)
	UCA1	*In vitro* (MCF-7: tamoxifen sensitive and LCC2: tamoxifen resistant cells)	Tamoxifen	Reduces the level of cleaved caspase 3, decreasing apoptosis and increasing cell viability	Xu et al. (2016)
	HISLA	*In vitro* (tumor tissues, MMDA‐MB‐231 cells)	Docetaxel, neoadjuvant chemotherapy	Binds to PHD2, preventing the interaction of PHD2-HIF-1a and thus inhibits the hydroxylation and degradation of HIF-1a, which leads to the increase of aerobic glycolysis. Protects tumor cells from apoptosis	Chen et al. (2019b)
	H19	*In vitro* (serum samples, MCF‐7 and MDA‐MB‐231 cells)	Doxorubicin	Increases cell viability and colony formation ability and decreases apoptosis	Wang et al. (2020)
	AGAP2-AS1	*In vitro* (SKBR-3 and BT474 cells)	Trastuzumab	Attenuates trastuzumab-induced cytotoxicity and apoptosis	Zheng et al. (2019)
		*In vitro* (serum samples, SKBR-3 and BT474 cells)	Trastuzumab	Induces trastuzumab resistance via modulating ATG10 expression and autophagy activity	Qian et al. (2021)
Ovarian	UCA1	*In vitro* (SKOV-3, A2780: cisplatin sensitive, SKOV-3-DDP, and A2780-DDP: cisplatin resistant cells)	Cisplatin	Regulates miR-143/FOSL2 signaling pathway to modulate cell growth and cisplatin resistance	Li et al. (2019d)
Cervical	HNF1A-AS1	*In vitro* (Hela/S: cisplatin sensitive and Hela/DDP: cisplatin resistant cells)	Cisplatin	Acts as a ceRNA for miR-34b to promote the expression of TUFT1 promoting proliferation and inhibition of apoptosis	Luo et al. (2019)

## 9 Potential therapeutic approaches

### 9.1 Targeting exosomal lncRNAs for therapy

Knowing that the expression of lncRNAs undergoes significant changes during tumorigenesis, and based on studies on lncRNAs involvement in carcinogenesis, the application of lncRNAs for diagnostic and therapeutic purposes seems necessary. LncRNAs can accumulate in exosomes and are present in different body fluids, tissues and cells (Hao et al., 2022; Zhang et al., 2021). In addition, exosomes are known as the third carriage after circulating tumor DNAs (ctDNAs) and circulating tumor cells (CTCs) in liquid biopsy and can be used as non-invasive biomarkers for cancer treatment (Zhang et al., 2021; Li et al., 2021a). The presence of some exosomal lncRNAs is an indication of tumor stage, treatment outcome (Lee et al., 2019), metastasis (Gao et al., 2018), tumor recurrence, or drug resistance (Zhang et al., 2021), and based on these results, it may be possible to plan a more fitting treatment for the affected person.

The emergence of immunotherapy, especially immune checkpoint inhibitors (ICIs), has become a revolution in cancer treatment. ICIs target immune receptors such as programmed cell death 1 (PD-1), programmed death ligand 1 (PD-L1), and cytotoxic T-lymphocyte-associated antigen 4 (CTLA-4) and enhance anticancer properties (Jacob et al., 2023). Exosomal lncRNAs have the ability to modulate the interaction between immune ligand and receptor, thereby bypassing the immune response of immune cells in different types of cancer (Zhang et al., 2022). The exosomal lncRNA KCNQ1OT1 secreted by colorectal cancer (CRC) cells inhibits ubiquitination of PD-L1 on CD8^+^ T cells by regulating the miR-30a-5p/USP22 pathway and escapes the immune response (Xian et al., 2021). Another study revealed that secretion of exosomes containing PCED1B-AS1 lncRNA from HCC in the co-culture of HCC cell line and human T cells can enhance the expression of PD-Ls via sponging hsa-mir-194-5p (Fan et al., 2021). According to studies, targeting exosomal lncRNAs involved in the regulation of immune checkpoints with siRNAs may be a potential therapeutic approach to reduce tumor progression (Zhang et al., 2022). For example, knockdown of the exosomal lncRNA PCAT6 in non-small cell lung cancer (NSCLC) using siRNA can suppress M2 polarization via the miR-326/KLF1 axis and extend tumor growth (Chen et al., 2022).

### 9.2 Current research and clinical trials focusing on exosomal lncRNAs

As mentioned previously, exosomal long non-coding RNAs have the potential to serve as valuable diagnostic, therapeutic, and prognostic biomarkers (Zhang et al., 2021; Da et al., 2021).

#### 9.2.1 Diagnostic markers

LY et al. found that exosomal lncRNA UEGC1 is highly expressed in patients with early-stage gastric cancer (EGC) compared with healthy individuals and has the potential to be as a sensitive noninvasive biomarker for EGC diagnosis (Lin et al., 2018). In another investigation, exosomal CRNDE-h was shown to be upregulated in the serum of patients with colorectal cancer (CRC) and derived from tumor cells. Based on the receiver operating characteristic (ROC) curve at a cut-off value of 0.020, the AUC reached a value of 0.892, with corresponding sensitivity and specificity rates of 70.3% and 94.4%, respectively, for the diagnosis of CRC (Liu et al., 2016b). According to one study, patients with early-stage NSCLC had significantly lower expression of growth arrest-specific transcript 5 lncRNA in the exosome (Exo-GAS5) compared with the control group. In terms of ROC curve analysis, Exo-GAS5 may have the potential to discriminate against individuals with stage I NSCLC and have an AUC value of 0.822 (Li et al., 2019e). RP11-85G21.1 (lnc85) is a novel exosomal lncRNA that was significantly elevated in HCC patients regardless of their alpha-fetoprotein (AFP) status and might be a potential diagnostic marker for HCC. RP11-85G21.1 was also shown to enhance HCC proliferation and migration via modulation of miR-324-5p (Huang et al., 2020). Plasma-derived exosomal SOX2-OT exhibited substantially increased expression levels in patients diagnosed with LSCC. This study suggests that SOX2-OT as a detecting biomarker can significantly differentiate LSCC patients from non-LSCC patients with an AUC of 0.864 in ROC analysis as a detecting biomarker (Teng et al., 2019). The study conducted by Abbastabar et al. demonstrated a significant upregulation of exosomal PVT-1, ANRIL, and PCAT-1 in urine samples from patients with bladder cancer (BC) compared with the control group. These findings suggest that these biomarkers hold potential as valuable diagnostic indicators for bladder cancer (Abbastabar et al., 2020).

#### 9.2.2 Prognostic markers

The independent prognostic factor of EOC was identified through a multivariate Cox regression model, which demonstrated that serum exosomal hypoxia-inducible factor (aHIF) level played a significant role. EOC patients were found to have elevated levels of exosomal aHIF in serum, and there was a positive correlation between the expression of serum exosomal aHIF and the aHIF lncRNA levels in EOC tissues. Furthermore, Kaplan-Meier survival analysis showed that EOC patients who had elevated expression of exosomal aHIF in serum experienced a significantly worse overall survival outcome (Tang et al., 2019b). According to another study, serum exosomal myocardial infarction associated transcript (MIAT) levels were remarkably upregulated in patients diagnosed with gastric cancer (GC) in comparison to individuals with gastric adenoma and healthy controls. The overall survival rate of GC patients with high expression of exosomal lncRNA aHIF in serum was lower, and MIAT was identified as an effective diagnostic and prognostic marker of GC (Xu et al., 2020b). The findings of another study revealed a correlation between the level of exosomal ATB and both tumor stage and portal vein thrombosis. Moreover, patients with elevated levels of circulating exosomal lncRNA-activated by tumor growth factor-beta (TGF-β) (lncRNA-ATB) exhibited notably reduced overall survival and progression-free survival rates (Lee et al., 2019). In addition, other exosomal lncRNAs such as CRNDE-h (Liu et al., 2016b), SPINT1-AS1 (Li et al., 2018b) and lncRNA 91H (Gao et al., 2018) in CRC, RP5-977B1 in NSCL (Min et al., 2022), and MALAT1 (Qiu et al., 2018) in OC have been identified as potential prognostic biomarkers.

#### 9.2.3 Treatment markers

Chen et al. demonstrated in their study that exosomal lncRNA lymph node metastasis-associated transcript 2 (LNMAT2), plays a significant role in promoting tube formation and migration of human lymphatic endothelial cells, as well as enhancing tumor lymphangiogenesis and lymph node (LN) metastasis in bladder cancer (BCa). The results suggested that LNMAT may be a treatment biomarker for LN metastasis in BCa (Chen et al., 2020a). Another investigation showed that exosomal H19 promoted resistance to erlotinib in NSCLC through the regulation of miR-615-3p/ATG7 axis which may offer a prospective diagnosis and treatment marker for NSCLC patients (Pan and Zhou, 2020). Furthermore, exosomal lncRNA HOTAIR, UCA1 and H19 in drug-resistant breast cancer and exosomal lncRNA UCA1 in drug-resistant ovarian cancer seem to be promising diagnostic biomarkers and molecular targets to reduce drug resistance (Tang et al., 2019a; Xu et al., 2016; Wang et al., 2020; Li et al., 2019d).

### 9.3 Strategies to overcome lncRNA-mediated drug resistance

Since exosomes are great nanoparticles for carrying cargoes such as ncRNAs, proteins, lipids, and other biological compounds, they can be used to carry lncRNAs capable of reversing drug resistance (Li et al., 2016) or anti-lncRNAs to knock down lncRNAs that cause drug resistance (Guo et al., 2020c; Wang et al., 2018). Applying various physical and chemical techniques, including electroporation, ultrasound, and liposome-mediated membrane fusion, can be utilized to acquire gene-enriched exosomes. Engineered exosomes can be modified to target specific tissues or organs, allowing for real-time monitoring of their pharmacokinetics and potentially improving therapeutic outcomes. This offers promising prospects for enhancing the efficacy of exosomal lncRNAs in clinical application (Yuan and Huang, 2021). Another approach could be using locked nucleic acids (LNAs), antisense oligonucleotides (ASOs), Small interfering RNA (siRNAs), CRISPR/Cas9 system, and some small designed molecules to disrupt exosomal lncRNAs responsible for anticancer drug resistance (Qu et al., 2016; Ye et al., 2022; Yousefi et al., 2020; Deocesano-Pereira et al., 2019). For instance, exosomal lncRNA lncARSR was upregulated in Sunitinib-Resistant RCC Cells, which acts as an oncogene and promotes sunitinib resistance through targeting miR-34/miR-44 to activate AXL and c-MET expression in RCC cells. It was also shown that utilization of locked nucleic acids (LNAs) that specifically target lncARSR might resensitize RCC cells to sunitinib (Qu et al., 2016). In a study that aimed to knockout MEG3 in Hs578T cells, the CRISPR/Cas9 system was performed. MEG3 deletion promoted cell proliferation, and cell growth (Deocesano-Pereira et al., 2019). Additionally, the regulation of lncRNAs can be achieved using specific small molecules. Hao et al. designed and identified a curcumin analogue called Comp34 which was able to suppress NUDT3-AS4 and consequently reduced the TNBC development. This compound effectively inhibited breast cancer by suppressing the lncRNA NUDT3-AS4 that acts as a sponge for miR-99s and increases the AKT1/mTOR pathway (Hao et al., 2020).

**FIGURE 6:**
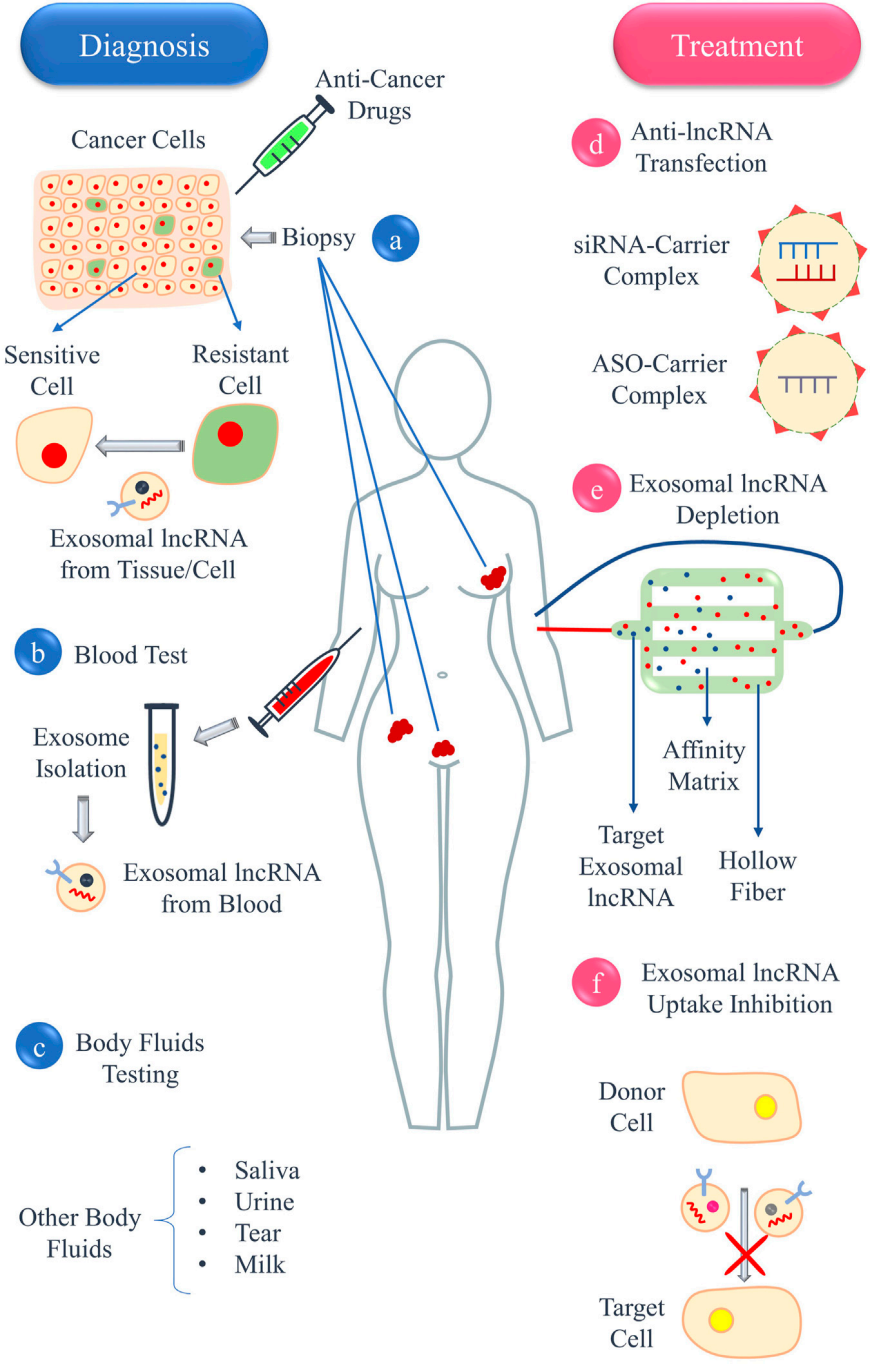
Application of exosomal lncRNAs in the diagnosis and treatment of female reproductive system cancers. Exosomal lncRNAs can be present in **(A)** tissue biopsy of cancer cells, **(B)** blood, or **(C)** other body fluids, such as saliva, urine, tears, and milk. Treatment methods include **(D)** using anti-lncRNAs, such as siRNAs or ASOs, against exosomal lncRNAs responsible for drug resistance, **(E)** depleting blood from exosomes containing lncRNAs involved in drug resistance using a system called ADAPT, and **(F)** inhibiting the uptake of exosomal lncRNAs by drug-sensitive cells.

Another strategy could be purifying blood from the exosomes containing lncRNAs involved in drug resistance (Kalluri, 2016). An example of this approach is the new system developed by Aethlon Medical called Aethlon ADAPTTM, which is capable of rapid absorption and selective retention of target particles smaller than 200 nm from the entire circulatory system. This technology is based on the interactions between serum components and an affinity matrix which contains substrates such as monoclonal antibodies and lectins in order to absorb tumor-derived exosomes and other oncological agents (Marleau et al., 2012). Moreover, prevention of exosomal lncRNA uptake by sensitive cells may be another solution (Xie et al., 2019; Marleau et al., 2012). Enhancing the effectiveness of therapy and impeding the progression of cancer can be achieved by developing drugs that hinder the transfer of exosomal lncRNAs from drug-resistant cells to drug-sensitive cells (Zhang et al., 2021). Undoubtedly, developing such therapeutic mechanisms requires more concentrated studies. The Combination of antitumor drugs and targeting involved exosomal lncRNAs can improve therapy outcomes and prevent tumor recurrence. Applications of exosomal lncRNAs in the diagnosis and treatment of female reproductive system cancers is summarized in Figure 6.

## 10 Challenges and future directions

### 10.1 Technical challenges in studying exosomal lncRNAs

Exosomal lncRNAs have plenty to offer when it comes to diagnostic methods, due to the fact that they exist in most body fluids and are secreted from almost every living cell. Nevertheless, there are still some potential circumscriptions in their clinical application as tumor markers (Hosseini et al., 2022; Yu et al., 2021). For example, exosome isolation methods are still far from being perfect. They can be extremely costly, difficult, and time-consuming, in addition to not being effective and sharp enough to provide reliable analytical results (Hosseini et al., 2022). One major problem in isolating exosomes is the contamination caused by ribonucleoprotein complexes, lipoproteins, and other contaminating molecules, and if the RNA levels are low, biological contamination can effortlessly interfere with the isolation process and create false results. Ultracentrifugation is probably the most prominent method for exosome isolation providing the highest purity (Hosseini et al., 2022; Xiao et al., 2020b; Makler and Asghar, 2020; Wu et al., 2020). Another significant step in studying exosomal lncRNAs is the identification and quantification of exosomes (Hosseini et al., 2022; Makler and Asghar, 2020). To precisely separate tumor signals from normal cell signals is a serious challenge. This problem is worsened when the liquid biopsy is done from plasma. A great volume of platelet-derived RNAs are present in plasma that can meddle with the results. Thus, employing tumor-specific surface markers to differentiate tumor-derived exosomes is of great importance (Yu et al., 2021; Brinkman et al., 2020). A few methods are being used at the moment in this regard. Flow cytometry, for example, is able to detect and classify exosomes by species and in great numbers. However, it requires expensive equipment, and the results it presents are not consistent. In enzyme-linked immunoassay (ELISA), specific antibodies against tumor markers are used. This allows ELISA to obtain tumor-derived exosomes in a more specific manner. Although this method seems very promising, in exosome identification with ELISA the samples are very prone to be contaminated with biological waste or other biomolecules. Another approach to identify exosomes is nanoparticle tracking analysis (NTA), whose limitations are its expensive equipment and time-consuming process (Hosseini et al., 2022; Makler and Asghar, 2020). The next technical challenge would be RNA extraction. Eldh et al. conducted a research to examine which RNA extraction method is best suited for lncRNAs. Seven different methods were investigated. The quality of lncRNAs were pretty high in all seven methods, but total RNA yield, size of RNA molecules, and purity of RNA varied in accordance to the method (Eldh et al., 2012). These results imply that choosing the appropriate method for extracting lncRNAs should be directed towards the aims of a research (Tellez-Gabriel and Heymann, 2019). At last, when it comes to detection of exosomal lncRNAs, the methods being used are mostly next-generation sequencing (NGS), lncRNA microarray, or RT-qPCR. New methods like dPCR have also been practiced, but highest levels of accuracy are yet to be achieved. NGS and lncRNA microarray are both considered expensive methods and they require big storage spaces for the data they generate; therefore, although there is room for improvement, RT-qPCR remains as the most common practice in lncRNA detection (Wu et al., 2020; Tellez-Gabriel and Heymann, 2019).

### 10.2 The future of personalized medicine considering exosomal lncRNA profiles

The study of lncRNAs have gained much interest through recent years, and while it appears to be a fairly promising approach in personalized medicine, there is still a lot to investigate and achieve in this field. Identifying pivotal lncRNAs by searching liquid biopsies or tissues from more patients and discovering their underlying mechanisms, the true nature of interactions between donor cells and recipient cells and how to minimize their undesirable communications, why some certain lncRNAs are chosen to enter exosomes and transfer to other cells, and how exosomes and their biological content affect each other are just a few of the perspectives that are yet to be fully comprehended (Yuan and Huang, 2021). For example, an interesting connection between expression levels of lncRNAs and the amount of exosomes a cell releases has been detected in some studies. In a 2019 study, Yang et al. observed that exosome secretion from HCC cells are elevated in response to lncRNA HOTAIR upregulation (Yang et al., 2019), and some other studies have demonstrated the opposite of this relationship (Li et al., 2021b; Xing et al., 2020). The heterogeneity of exosomes is another intriguing subject to explore. Tumors can secrete a variety of exosome subtypes. Identifying and categorizing these subtypes like how blood cells are classified, and understanding how lncRNAs are loaded into each subtype of exosomes can be a big step in using exosome-derived lncRNAs as a mean of personalized tumor therapy (Hosseini et al., 2022; Xiao et al., 2020b; Wu et al., 2020).

Aside from further understanding the molecular aspects of exosomal lncRNAs, the techniques and equipment used in this regard hold great importance. One important factor to consider when studying and using exosomes and its content as biomarkers is the methods that are used to purify exosomes. It has been observed that exosomal lncRNAs obtained from different cells and patients vary in biological and functional aspects. Accordingly, it seems reasonable that methods used to isolate exosomes and their cargos should be performed with precision and under uniform for them to have clinical application. The study in this field is still in its beginning and to better understand and compare the results, it is important that the methods are standardized so no problem is caused during the normalization of results (Yuan and Huang, 2021; Xiao et al., 2020b). To choose the optimum method, the problem being addressed in a research should be considered. For instance, when an exosomal lncRNA is investigated as a biomarker to diagnose a disease and its relationship with the disease has already been proven and clear, the purity of exosomes is not as important. However, when it comes to determining the relationship between an exosomal lncRNA and other biological molecules, molecular pathways, or diseases, it is important to prevent any false result and inaccuracy; thus, applying a separation method that provides samples with the highest levels of purity seems like a wise approach (Wu et al., 2020). That being said, it is clear that exosomal lncRNA utility in personalized medicine is still in its infancy. Thus, for it to achieve real clinical application, a long track of investigations and innovative approaches is ahead.

## 11 Conclusion

Several studies on lncRNAs support the fact that they have regulatory functions in numerous molecular processes. These molecules are widely detected in different tissues, cells, and body fluids. Since lncRNAs can be transferred from 1 cell to another through exosomes, they can exert their regulatory actions in new areas. Exosomal lncRNAs by being involved in various biological processes such as migration, invasion, proliferation, and drug resistance can modulate the progression of cancer. The mechanisms of drug resistance in reproductive cancers, including breast, ovarian, and cervical cancers, were found to be highly influenced by the regulatory actions of exosome-derived lncRNAs. Hence, a comprehensive understanding of the intricate mechanisms involved in the cancer microenvironment, such as exosomal lncRNAs, which are the focus of this study, is crucial for the effective treatment of these cancers. According to several investigations exosomal lncRNAs show promise as potential diagnostic, prognostic, and treatment biomarkers for predicting drug resistance and monitoring drug efficacy. By elucidating the mechanisms underlying drug resistance through exosomal lncRNAs and related research, as well as discussing treatment strategies and associated challenges, this study provides a broad perspective for designing future studies in the field of targeted cancer treatment and potential drug development. Given that we possess little knowledge concerning exosomal lncRNAs, the need for more fundamental research and improvement in methods used in this field seems evident. It is necessary to fully understand and identify key exosome-derived lncRNAs and uncover the exact mechanisms affected by them. Achieving this data is a major step in using and targeting lncRNAs clinically. For example, if lncRNAs are to be used for diagnosis purposes, the identification of universal lncRNA biomarkers should be earnestly pursued. Same exosomal lncRNAs have been released and identified in different malignancies. This can cause challenges in the process of using lncRNAs as specific biomarkers or their clinical application. Undoubtedly, further comparative studies with large sample sizes are required to solve these problems (Xiao et al., 2020b; Kang et al., 2022). As for the techniques and equipment that are currently available, although a lot of them showcase precision and efficiency, a sound method is yet to be introduced (Wu et al., 2020). High accuracy, low cost, simple procedure, and time efficiency are some of the factors that should be taken into account for developing new methods (Hosseini et al., 2022). The need for more methods applicable for isolation, identification and classification of exosomes, RNA extraction, and analysis of exosomal lncRNAs with high-throughput has never been greater (Wu et al., 2020). Bearing in mind that the study of exosomal lncRNAs is in its early days and there is a lot to be discovered about them, this field of study should be paid more attention and effort.
